# Towards next generation antisense oligonucleotides: mesylphosphoramidate modification improves therapeutic index and duration of effect of gapmer antisense oligonucleotides

**DOI:** 10.1093/nar/gkab718

**Published:** 2021-08-20

**Authors:** Brooke A Anderson, Graeme C Freestone, Audrey Low, Cheryl L De-Hoyos, William J Drury III, Michael E Østergaard, Michael T Migawa, Michael Fazio, W Brad Wan, Andres Berdeja, Eli Scandalis, Sebastien A Burel, Timothy A Vickers, Stanley T Crooke, Eric E Swayze, Xuehai Liang, Punit P Seth

**Affiliations:** Ionis Pharmaceuticals, 2855 Gazelle Court, Carlsbad, CA 92010, USA; Ionis Pharmaceuticals, 2855 Gazelle Court, Carlsbad, CA 92010, USA; Ionis Pharmaceuticals, 2855 Gazelle Court, Carlsbad, CA 92010, USA; Ionis Pharmaceuticals, 2855 Gazelle Court, Carlsbad, CA 92010, USA; Ionis Pharmaceuticals, 2855 Gazelle Court, Carlsbad, CA 92010, USA; Ionis Pharmaceuticals, 2855 Gazelle Court, Carlsbad, CA 92010, USA; Ionis Pharmaceuticals, 2855 Gazelle Court, Carlsbad, CA 92010, USA; Ionis Pharmaceuticals, 2855 Gazelle Court, Carlsbad, CA 92010, USA; Ionis Pharmaceuticals, 2855 Gazelle Court, Carlsbad, CA 92010, USA; Ionis Pharmaceuticals, 2855 Gazelle Court, Carlsbad, CA 92010, USA; Ionis Pharmaceuticals, 2855 Gazelle Court, Carlsbad, CA 92010, USA; Ionis Pharmaceuticals, 2855 Gazelle Court, Carlsbad, CA 92010, USA; Ionis Pharmaceuticals, 2855 Gazelle Court, Carlsbad, CA 92010, USA; Ionis Pharmaceuticals, 2855 Gazelle Court, Carlsbad, CA 92010, USA; Ionis Pharmaceuticals, 2855 Gazelle Court, Carlsbad, CA 92010, USA; Ionis Pharmaceuticals, 2855 Gazelle Court, Carlsbad, CA 92010, USA; Ionis Pharmaceuticals, 2855 Gazelle Court, Carlsbad, CA 92010, USA

## Abstract

The PS modification enhances the nuclease stability and protein binding properties of gapmer antisense oligonucleotides (ASOs) and is one of very few modifications that support RNaseH1 activity. We evaluated the effect of introducing stereorandom and chiral mesyl-phosphoramidate (MsPA) linkages in the DNA gap and flanks of gapmer PS ASOs and characterized the effect of these linkages on RNA-binding, nuclease stability, protein binding, pro-inflammatory profile, antisense activity and toxicity in cells and in mice. We show that all PS linkages in a gapmer ASO can be replaced with MsPA without compromising chemical stability and RNA binding affinity but these designs reduced activity. However, replacing up to 5 PS in the gap with MsPA was well tolerated and replacing specific PS linkages at appropriate locations was able to greatly reduce both immune stimulation and cytotoxicity. The improved nuclease stability of MsPA over PS translated to significant improvement in the duration of ASO action in mice which was comparable to that of enhanced stabilized siRNA designs. Our work highlights the combination of PS and MsPA linkages as a next generation chemical platform for identifying ASO drugs with improved potency and therapeutic index, reduced pro-inflammatory effects and extended duration of effect.

## Graphical Abstract

**Graphical Abstract:**
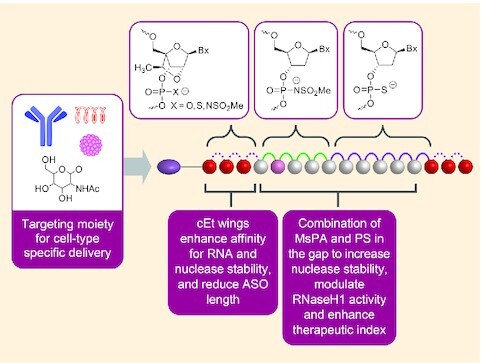
Towards next generation antisense oligonucleotides: mesylphosphoramidate modification improves therapeutic index and duration of effect of gapmer antisense oligonucleotides.

## INTRODUCTION

**Figure 1.**
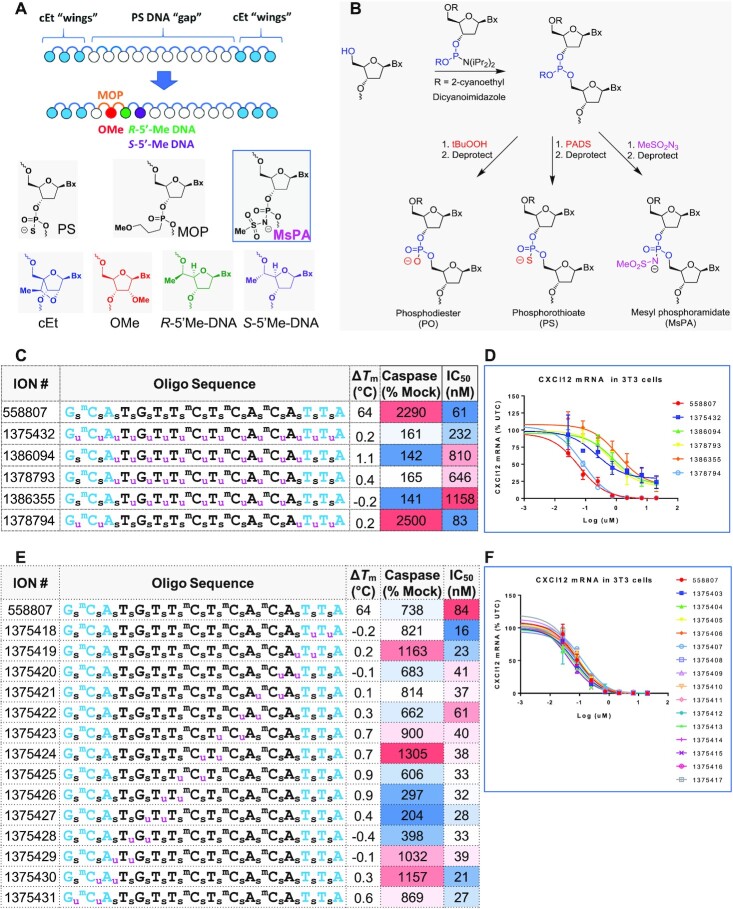
MsPA enhances the therapeutic index of PS gapmer ASOs. (**A**) Design of gapmers and gap-modifications that enhance therapeutic index. (**B**) Synthesis of PO, PS and MsPA ASOs. (**C**) Design, Tm, cytotoxicity and antisense activity of ASOs with full MsPA in gap and/or wings. (**D**) Dose-response curves for reducing CXCl12 mRNA in NIH 3T3 cells. (**E**) Design, Tm, cytotoxicity and antisense activity of ASOs with two MsPA walked across length of ASO. (**F**) Dose-response curves for reducing CXCl12 mRNA in NIH 3T3 cells. Blue letters indicate cEt, black DNA, subscript u = MsPA, s = PS, superscript m = 5-Me group on cytosine nucleobases.

The phosphorothioate (PS) linkage represents a privileged modification in oligonucleotide therapeutics (1). The PS linkage is critical for enhancing the drug-like properties of single stranded gapmer antisense oligonucleotides (ASOs) that promote RNaseH1-mediated degradation of RNA (2) (Figure 1A). PS helps stabilize the DNA ‘gap’ region from nucleolytic degradation and is one of very few backbone modifications that support RNaseH1 activity. In addition, PS is also used to stabilize siRNA therapeutics from exonuclease degradation resulting in durable clinical responses (3).

A unique feature of PS ASOs is their ability to bind many proteins with micromolar to nanomolar dissociation constants (4). The anionic sulfur in PS linkages can interact with amino acid side-chains in proteins by electrostatic and hydrophobic interactions and typically 10–13 PS linkages in single stranded oligonucleotides are required to establish substantial binding (5). Significant progress has been made in the last decade in our understanding of how PS ASOs interact with plasma, cell-surface and intracellular proteins leading to the discovery of more effective antisense agents (6). Furthermore, we also recently defined the molecular mechanisms of cytotoxic PS ASOs, characterized the major steps in the pathway and demonstrated that straightforward chemical approaches that modified specific sites in toxic PS ASOs could reduce or ablate cyto-toxicity (7–10) (Figure 1A). However, the non-specific protein binding of some PS ASOs can also cause pro-inflammatory effects, species specific complement activation and thrombocytopenia (11). While these effects can be mitigated by judicious screening and evaluation of candidate ASOs in pre-clinical cell and animal models, a general chemical strategy to modulate ASO protein binding by reducing PS content could be beneficial for designing the next generation of more effective PS ASOs.

The mesylphosphoramidate (MsPA) linkage was recently reported by Stetsenko as an alternate to PS for RNaseH1-mediated knock down of micro RNA targets and to modulate mRNA splicing (12–14). In the MsPA linkage, one of the non-bridging oxygen atoms in the phosphodiester linkage is replaced with methanesulfonylamido group (Figure 1A). This linkage differs from phosphoramidate and alkylphosphonate linkages in that it retains negative charge on the phosphate backbone. However, it lacks the negatively charged sulfur atom that is the primary pharmacophore for ASO-protein interactions (15). We hypothesized that a combination of MsPA and PS linkages in gapmer ASOs could modulate ASO-protein interactions and further improve the therapeutic properties of PS gapmer ASOs.

In this manuscript, we evaluated the effect of introducing MsPA linkages in the DNA gap and flanks of gapmer ASOs and characterized the effect of these linkages on RNA-binding, nuclease stability, protein binding, pro-inflammatory profile, antisense activity and toxicity in cells and in mice. We show that all PS linkages in a gapmer ASO can be replaced with MsPA without compromising chemical stability and RNA binding affinity but these designs generally reduced activity. However, replacing up to 5 PS in the gap with MsPA maintained, and in some cases, improved potency. Furthermore, replacing 3–5 PS linkages near the 5′-side of the gap greatly reduced cytotoxicity of model toxic ASOs. The combination of MsPA and PS reduced non-specific protein binding resulting in reduced nuceolar protein misclocalization and mitigated cytokine induction in BJAB cells. Lastly, the increased nuclease stability of the MsPA linkage translated to a longer duration of pharmacodynamic effect in mouse models that was comparable to an enhanced stabilized siRNA versus the same gene target. Our work highlights the combination of PS and MsPA linkages as a next generation chemical platform for identifying ASO drugs with improved potency and safety, reduced pro-inflammatory effects and extended duration of effect in the clinic.

## MATERIALS AND METHODS

### Synthesis of MsPA ASOs

Oligonucleotides were synthesized on a 2 μmol scale using Nittophase UnyLinker support (200 μmol/g) on an ABI 394 DNA/RNA synthesizer. Fully protected nucleoside phosphoramidites were incorporated using standard solid-phase oligonucleotide synthesis, i.e. 3% dichloroacetic acid in dichloromethane for deblocking, 1 M 4,5-dicyanoimidazole 0.1 M *N*-methylimidazole in acetonitrile as activator for phosphoramidite couplings, 20% acetic anhydride in THF and 10% 1-methylimidazole in THF/pyridine for capping and 0.1 M xanthane hydride in pyridine:acetonitrile 3:2 (v:v) for thiolation. MsPA couplings were oxidized instead of thiolated using 0.5 M mesyl azide in acetonitrile:toluene 1:1 (v:v) with oxidation times varying depending on the steric hindrance of the phosphoramidite being oxidized (e.g. 3 × 500 s for DNA oxidations and 6 × 900 s for cEt oxidations). Phosphoramidites were dissolved to 0.1 M in acetonitrile:toluene 1:1 (v:v) and incorporated using 6 min coupling time for DNA phosphoramidites and 10 min for all other phosphoramidites. At the end of the solid phase synthesis cyanoethyl protecting groups were removed by a 30 min treatment with 20% diethylamine in toluene. ASOs were deprotected and cleaved using conc. aq. ammonia at room temperature for 48 h or at 55°C overnight.

Chiral MsPA oligonucleotides were synthesized on an ABI 394 DNA/RNA synthesizer. They were made on a 2 μmol scale using polystyrene-based NittoPhase UnyLinker support (405 μmol/g). Commercially available fully protected nucleoside phosphoramidites were used for the stereo-random incorporations and stereodefined oxazaphospholidine (OAP) monomers (16) for the chiral MsPA incorporation. Standard conditions were used for the synthesis, i.e. 3% DCA in DCM for deblocking; 1.0 M 4,5-dicyanoimidazole and 0.1 M *N*-methylimidazole in acetonitrile as activator, 0.15 M DNA phosphoramidite in acetonitrile (5 min coupling time); Cap A: acetic anhydride in THF and Cap B: 10% 1-methylimidazole in THF/pyridine for capping. Oxidations of the P(III) species were performed as followed: 10% tert-butyl hydroperoxide in acetonitrile (2 × 5 min) for PO, 0.1 M xanthane hydride in 1:1 pyridine:acetonitrile (2 × 3 min) for PS, and 0.5 M mesylazide in 1:1 MeCN:toluene (6 × 15 min) for stereo-random and chiral MsPA. After synthesis, the cyanoethyl protecting groups were removed using 1:1 triethylamine:acetonitrile solution and the remaining protecting groups were removed using conc. aq. ammonia at 85°C for 1 h.

Oligonucleotides were isolated by HPLC using a combined purification, detritylation, desalt method. During the basic RSR (Reverse phase, SAX, Reverse phase) method the sample is loaded onto the RP column in H_2_O. A failure elution is then performed on the RP column with 1:1 (A: 80% MeOH/water, B: 2.5 M NaCl, 50 mM NaOH). DMT cleavage is then performed on the RP column with 6% DCA, followed by a water wash. Next, the detritylated compound is loaded onto the SAX column with 80% MeOH. The RP column is equilibrated with 50 mM NaOH. A SAX gradient is then performed from 0 to 50% with A and B buffers (A: 50 mM NaOH, B: 50 mM NaOH, 2.5 M NaCl) Once a UV absorbance threshold is reached, the compound is then loaded back onto the RP column. 250 mM NaCl is flushed through the RP column for cation exchange, water is flowed through the column for desalt, and the final compound is eluted in 1:1 MeCN:water.

### *T*_m_ measurements

ASO and RNA were mixed in 1:1 ratio (4 μM duplex) in a buffer containing 100 mM NaCl, 10 mM phosphate and 10 mM EDTA at pH 7. Oligos were hybridized with the complementary RNA strand by heating duplex to 85°C for 5 min and allowed to cool at room temperature. Thermal denaturation temperatures (*T*_m_ values) were measured in quartz cuvettes (pathlength 1.0 cm) on a Cary 100 ultraviolet (UV)/visible spectrophotometer equipped with a Peltier temperature controller. Absorbance at 260 nm was measured as a function of temperature using a temperature ramp of 0.5°C per min. *T*_m_ values were determined using the hyperchromicity method incorporated into the instrument software.

### Animal experiments

Animal experiments were conducted in accordance with the American Association for the Accreditation of Laboratory Animal Care guidelines and were approved by the Animal Welfare Committee (Cold Spring Harbor Laboratory's Institutional Animal Care and Use Committee guidelines). Animals were housed in micro-isolator cages on a constant 12-h light–dark cycle with controlled temperature and humidity and were given access to food and water ad libitum. 5–7 week old BALB/C mice (Charles Rivers Laboratory) were treated with a single subcutaneous injection. Seventy-two hours following treatment animals were sacrificed and blood and tissues were collected. Blood was collected by cardiac puncture exsanguination with K3-EDTA (SARSTEDT, Germany) and plasma transaminases were measured using a Beckman Coulter AU480 analyzer.

### ASO *in vitro* and *in vivo* activity assay

NIH3T3 cells were mixed with ASOs at the specified final concentrations in a final volume of 100 μl and added to a BTX high-throughput electroporation plate. The cells were then electroporated using the ECM 830 high-throughput electroporation system. Twenty-four hours after electroporation, total RNA was prepared using RNeasy mini Kit (Qiagen).

For the liver, 50–100 mg of liver tissue was homogenized with an Omni Tissue Homogenizer (Omni International) in guanidinium thiocyanate with 8% beta mercaptoethanol, and total RNA was isolated using the PureLink Pro 96 Total RNA Purification Kit (LifeTechnologies, Carlsbad, CA).

The targeted mRNA levels were quantified through quantitative real-time PCR (qRT-PCR) assay performed in triplicate using the StepOne Real-Time PCR system and TaqMan primer probe sets with either the AgPath-ID One-Step RT–PCR Reagents (Thermo Fisher Scientific) or the Express One-Step SuperMix qRT-PCR Kit (Life Technologies, Carlsbad, CA). Primers and probes for the PCR reactions were obtained from Integrated DNA technologies (IDT). The sequences for the primers and probe used for mouse CXCL12 are 5′- CCAGAGCCAACGTCAAGCAT-3′ for the forward primer, 5′- CAGCCGTGCAACAATCTGAA-3′ for the reverse primer, and 5′-TGAAAATCCTCAACACTCCAAACTGTGCC-3′ for the probe. Mouse Hdac2: 5′-TGATGGTGTTGAGGAAGCTTTTT-3′ for the forward primer, 5′-TCCCTCAAGTCTCCTGTTCCA-3′ for the reverse primer, and 5′-ACAACAGATCGCGTGATGACCGTCTC-3′ for the probe. Mouse Dynamin2: 5′-AGAGGAGACCGAGCGAAT-3′ for the forward primer, 5′-CATGGTTTGTGTTGATGTACGAC-3′ for the reverse primer, and 5′-CCTACATCAGGGAGCGAGAAGGGA-3′ for the probe. Mouse FXI: Forward: 5′-ACATGACAGGCGCGATCTCT-3′, 5′-TCTAGGTTCACGTACACATCTTTGC-3′ for the reverse and 5′-TTCCTTCAAGCAATGCCCTCAGCAAT-3′ for the probe. Mouse Pabn1: 5′-CCGGAGCTAGAAGCGATCAA-3′ for the forward primer, 5′-CCTTTAGCTTCTCAGCCTCTTCCT-3′ for the reverse primer, and 5′-CTCGAGTCAGGGAGATG-3′ for the probe. Mouse FXII: 5′-CAAAGGAGGGACATGTATCAACAC-3′ for the forward primer, 5′- CTGGCAATGTTTCCCAGTGA-3′ for the reverse primer, and 5′- CCCAATGGGCCACACTGTCTCTGC-3′ for the probe.

qRT-PCR data were analyzed with StepOne Software v.3 (Applied Biosystems). The expression levels of target RNAs were normalized to total RNA in duplicate RNA samples quantified using Quant-iT RiboGreen RNA Reagent (Thermo Fisher Scientific). IC50 was calculated using PRISM.

### Caspase 3*/*7 assays

ASOs were delivered into Hepa1-6 cells by electroporation at 20 μM and cells were incubated for 24 h. For quantitative analysis of caspase activation, Caspase-Glo 3*/*7 Reagent (Promega) was added directly to cells in a 96-plate at a volume equal to the sample volume. Luminescence was recorded after 30 min incubation using a TECAN Infinite M200 plate reader. Background readings determined from wells containing culture medium only were subtracted. Relative caspase activity was calculated as 100% × luminescence reading of a treated sample*/*a mock-treated control.

### Immunofluorescence staining

HeLa cells transfected with 120 nM ASOs for 5 h were fixed with 4% paraformaldehyde in PBS for 30 min, and permeabilized with 0.1% Triton in PBS for 4 min at room temperature. After incubation at room temperature for 30 min with block buffer (1 mg/ml BSA in PBS), cells were incubated at room temperature with P54nrb antibody (Santa Cruz Biotech. sc-376865, 1:100–1:300) in block buffer for 2 h, washed three times (5 min each) using wash buffer (0.1% NP-40 in 1 × PBS), and incubated for 1 h with AF488-conjugated anti-mouse secondary antibody (Abcam, ab150113, 1:200). After washing three times, cells were mounted with Anti-fade reagent containing DAPI (Life Technologies), and images were taken using confocal microscope (Olympus FV-1000) and processed with FV-10 ASW 3.0 Viewer software (Olympus).

### ASO-protein interaction using NanoBret assay

The constructs expressing the NLuc-fused model proteins were described previously (17,18). The expression plasmids were transfected into 6 × 10^5^ HEK 293 cells using Effectene transfection reagent (Qiagen) based on the manufacturer's protocol. 24-h later, cells were collected by trypsinization, washed once with 1× PBS, and resuspended in 250 μl Pierce IP Lysis Buffer (Thermo Scientific). Cell lysates were incubated 30 min at 4°C while rotating, then cleared by centrifugation at 15 000 rpm for 5 min. The fusion proteins were purified by adding 20 μl HisPur Ni-NTA Magnetic Beads (Thermo Scientific) and 10 mM imidazole then incubating at 4°C for 2 h. Beads were washed 4 times with 1× PBS, 10 mM imidazole, and 0.01% Tween-20. Fusion protein was eluted from the beads in 100 μl 1× PBS and 200 mM imidazole, followed by dilution with of 200 μl IP buffer.

BRET assays were performed in white 96-well plates as previously described (17). 10 nM Alexa-linked ASO 766634 and different concentrations of unconjugated competing ASOs were incubated at room temperature for 15 min in 1X binding buffer (100 mM NaCl, 20 mM Tris–HCl, pH 7.5, 1 mM EDTA and 0.1% NP-40) with 10^6^ RLU/well of Ni-NTA-purified NLuc fusion protein. Following the incubation, NanoGlo substrate (Promega) was added at 0.1 μl/well. Readings were performed for 0.3 sec using a Glomax Discover system using 450 nm/8 nm band pass for the donor filter, and 600 nm long pass for the acceptor filter. BRET was calculated as the ratio of the emission at 600/450 nm (fluorescent excitation emission/RLU).

### RNase H1 cleavage assay

Purified human RNase H1 was diluted in buffer containing 100 mM Tris–HCl, pH 7.4, 50 mM NaCl, 30% glycerol and 10 mM DTT. ASO*/*RNA duplex was formed with ASO and 5′-FAM-labeled complementary RNA at a final concentration of 0.33 μM each by annealing in reaction buffer containing 20 mM Tris–HCl, pH 7.4, 50 mM NaCl, 10 mM MgCl_2_ and 10 mM DTT. RNase H1 protein was added to the duplex at a final concentration of 1 ng per reaction and the reaction was performed at 37°C for 15 min. The reactions were stopped by adding 10 μl stop solution containing 8 M urea and 120 mM EDTA for every 20 μl of reaction mix. Samples were heated at 95°C for 5 min and separated on a 20% denaturing polyacrylamide gel, and cleavage products were visualized using a Storm PhosphorImager and analyzed with ImageQuantTL software.

### BJAB assay

Human BJAB cells (DSMZ – German Collection of Microorganisms and Cell Cultures GmbH) were cultured in RPMI + 20% FBS at 37°C and 5% CO_2_. Cells were routinely sub-cultured and maintained at a density of 0.1–1.5 × 10^6^ cells per ml. Optimal confluency and viability are seen when cells visibly clump together but media has not yet turned yellow. Cells are quantitated and diluted to 3.75 × 10^5^ cells per ml in growth medium plus 50 units/ml penicillin and 50 μg/ml streptomycin before plating 100 μl to the wells of a 96-well Costar v-bottom plate (Corning Ref # 3894). Immediately after plating the cells, 11 μl of 10× oligo or water is added to the wells of the cell plate. The cell plates are then incubated at 37°C and 5% CO_2_ for 24 h before collecting the cells and lysing for RNA purification and analysis.

The RNA was purified with a glass fiber filter plate (Pall 5072, VWR Radnor, PA) using chaotropic salts. The inflammatory potential of the compounds is determined through the quantitation of the human *CCL22* transcript expression. The human *CCL22* and *GAPDH* mRNA was quantitated with RT-qPCR on the QS7 instrument (Applied Biosystems, Foster City, CA) using the following primer probe sets (*CCL22* PP Set: For: CGCGTGGTGAAACACTTCTA, Rev: GATCGGCACAGATCTCCTTATC, Probe: TGGCGTGGTGTTGCTAACCTTCA and *GAPDH* PP Set: For: GAAGGTGAAGGTCGGAGTC, Rev: GAAGATGGTGATGGGATTTC, Probe: CAAGCTTCCCGTTCTCAGCC). Briefly, 5 μL RT-qPCR reactions containing 1 μL of RNA were run with Agpath-ID reagents and the primer probe sets following the manufacturers’ instructions. *GAPDH* expression levels were used to normalize the *CCL22* results. Data were analyzed using Microsoft Excel (v14.4, Microsoft, Redmond, WA).

### Duration of effect

5–7 week old male C57/B6J mice (Jackson Laboratories) were cared for and housed as previously mentioned. All blood sampling was done by submandibular bleeding with K3-EDTA collection tube (SARSTEDT, Germany). For the baseline collection, blood was collected 4–6 h prior to treatment with a single subcutaneous injection. Plasma was separated from the whole blood by centrifugation and stored at –80°C until the samples were run in the Mouse Factor XII total antigen assay ELISA kit (Molecular Innovations, Novi, MI) following the manufactures protocol to determine protein levels in the plasma.

## RESULTS

### Synthesis and duplex stabilizing properties of MsPA ASOs

The MsPA linkage was introduced into ASOs by means of a Staudinger reaction between methanesulfonyl azide (MsN_3_, 0.5 M solution in 1:1 acetonitrile/toluene) and the trivalent phosphite intermediate produced upon phosphoramidite coupling (19) (Figure 1B). The MsPA ASOs were stable during synthesis and deprotection and no by-products arising from cleavage at the sites of MsPA incorporation were observed in the HPLC and mass spectra of the crude reaction mixtures following deprotection with aqueous ammonia (Supplementary Figure S1). The MsPA ASOs were also obtained in yields and purities comparable to PS ASOs following purification by ion-exchange chromatography (Supplementary Figure S2).

All the MsPA ASOs were evaluated in thermal denaturation experiments versus complementary RNA (Figure 1C and E). The MsPA linkage was generally well tolerated and replacing two or all PS with MsPA did not reduce melting temperature of the ASO/RNA duplexes relative to the parent full PS ASO. These data suggest that the increased bulk of the MsPA linkage is well accommodated within the ASO/RNA duplex. In contrast, introducing even two methyl-phosphonate linkages was reported to result in significant duplex destabilization and chemical instability (10). Thus, sterically larger substituents (MsPA) that maintain the negative charge on the backbone phosphate may be less detrimental to duplex stability than small substituents that neutralize backbone charge (methyl-phosphonates).

### Determining optimal placement of MsPA in PS ASOs to improve therapeutic index

To determine the optimal positions for MsPA in PS ASOs to improve therapeutic index, we synthesized ASOs that were completely modified with MsPA or modified with MsPA in the gap, gap-junctions and the wings of a model toxic constrained ethyl BNA-modified (cEt) (20) gapmer ASO 558807 targeting CXCl12 mRNA. The MsPA ASOs were evaluated for antisense activity in NIH3T3 cells following delivery by electroporation. ASOs where all PS in the gap was replaced with MsPA (1375432, 1386094, 1378793, 1386355) showed reduced potency and cytotoxicity relative to the parent, model toxic ASO 558807, as determined by qRT-PCR quantification of the targeted CXCL12 mRNA levels and caspase activity assays, respectively (Figure 1C and D). In contrast, introducing MsPA in the cEt wings (1378794) did not reduce potency or cytotoxicity. Interestingly, ASOs fully modified with MsPA in the DNA gap also showed reduced efficacy (maximal knockdown) relative to the parent ASO. This suggests that full MsPA substitution does not support optimal RNase H1 activity, and that some PS DNA is needed to maximize both potency and efficacy.

We next determined if site-specific replacement of PS in the gap with MsPA could maintain potency and efficacy. Because our model sequence elicited cytotoxicity at higher doses, we were also able to study the effects of MsPA substitutions on cytotoxicity. We walked one or two MsPA in a row across the entire ASO and evaluated the modified ASOs in cells for CXCl12 mRNA knockdown. Introducing a single MsPA linkage had minimal to no effect on potency or cytotoxicity (Supplementary Figure S3). However, introducing two MsPA linkages had a more pronounced effect and significant reduction in cytotoxicity was observed when the MsPA linkage was introduced at positions 2, 3 (1375427) and 3, 4 (1375426) from the 5′-cEt/DNA gap-junction (Figure 1E and F). Importantly, all ASOs with 2 MsPA in the gap or cEt wings did not reduce the activity, rather, similar or improved potency was observed relative to the parent control ASO 558807.

### Controlling chirality of MsPA linkages does not enhance potency, therapeutic index or nuclease stability relative to racemic MsPA linkages

**Figure 2.**
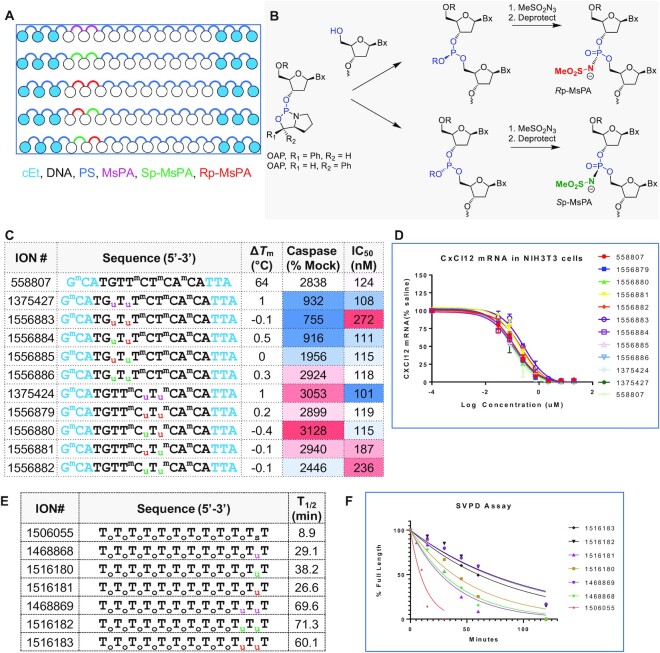
Controlling MsPA chirality is not beneficial for improving therapeutic index or nuclease stability. (**A**) Design of chiral MsPA gapmer ASOs evaluated. (**B**) Synthesis of chiral MsPA ASOs using OAP chemistry. (**C**) Design, Tm, cytotoxicity and antisense activity of ASOs with chiral MsPA linkages. Position of the MsPA linkages are shown while the rest of the ASO is stereorandom PS modified. (**D**) Dose-response curves for reducing CXCl12 mRNA in NIH3T3 cells. (**E**) Sequence and (**F**) evaluation of MsPA oligonucleotides for stability versus exonuclease digestion using snake venom phosphodiesterase (SVPD). Blue letters indicate cEt, black DNA, subscript pink u = MsPA, green u = Sp MsPA, red u = Rp MsPA, s = PS, o = PO, superscript m = 5-Me group on cytosine nucleobases.

Like the PS linkage, the MsPA linkage is chiral and can exist in the Sp or Rp configurations (Figure 2A) (21). To determine the effect of controlling chirality of MsPA linkages, we synthesized ASOs with RpRp, RpSp, SpRp and SpSp configurations at positions 2, 3 and positions 5, 6 in the DNA gap using the OAP chemistry pioneered by Wada (Figure 2B) (22). The MsPA linkage was configurationally stable and could be synthesized with high levels of stereoselectivity using this methodology (Supplementary Figure S4).

The MsPA ASOs were evaluated in thermal denaturation experiments where both isomers showed almost identical duplex stabilizing properties (Figure 2C). The MsPA ASOs were evaluated for antisense activity in NIH3T3 cells following delivery by electroporation. All ASOs showed almost identical potencies suggesting that chirality of the MPA linkage has minimal impact on antisense activity (Figure 2C and D). The MsPA ASOs were also evaluated in a cytotoxicity assay. ASOs with MsPA at positions 2, 3 mitigated cytotoxicity, however, controlling the chirality did not show beneficial effects. Furthermore, MsPA at positions 5 and 6 had a minimal impact on cytotoxicity (Figure 2C). We also evaluated the MsPA ASOs for enhancing exonuclease stability using snake venom phosphodiesterase. Both configurations of the MsPA linkage were equally efficient at stabilizing the oligo from 3′-to-5′ exonuclease digestion relative to racemic MsPA (Figure 2E and F).

### MsPA linkages modulate RNaseH1 cleavage patterns on ASO/RNA heteroduplexes

**Figure 3.**
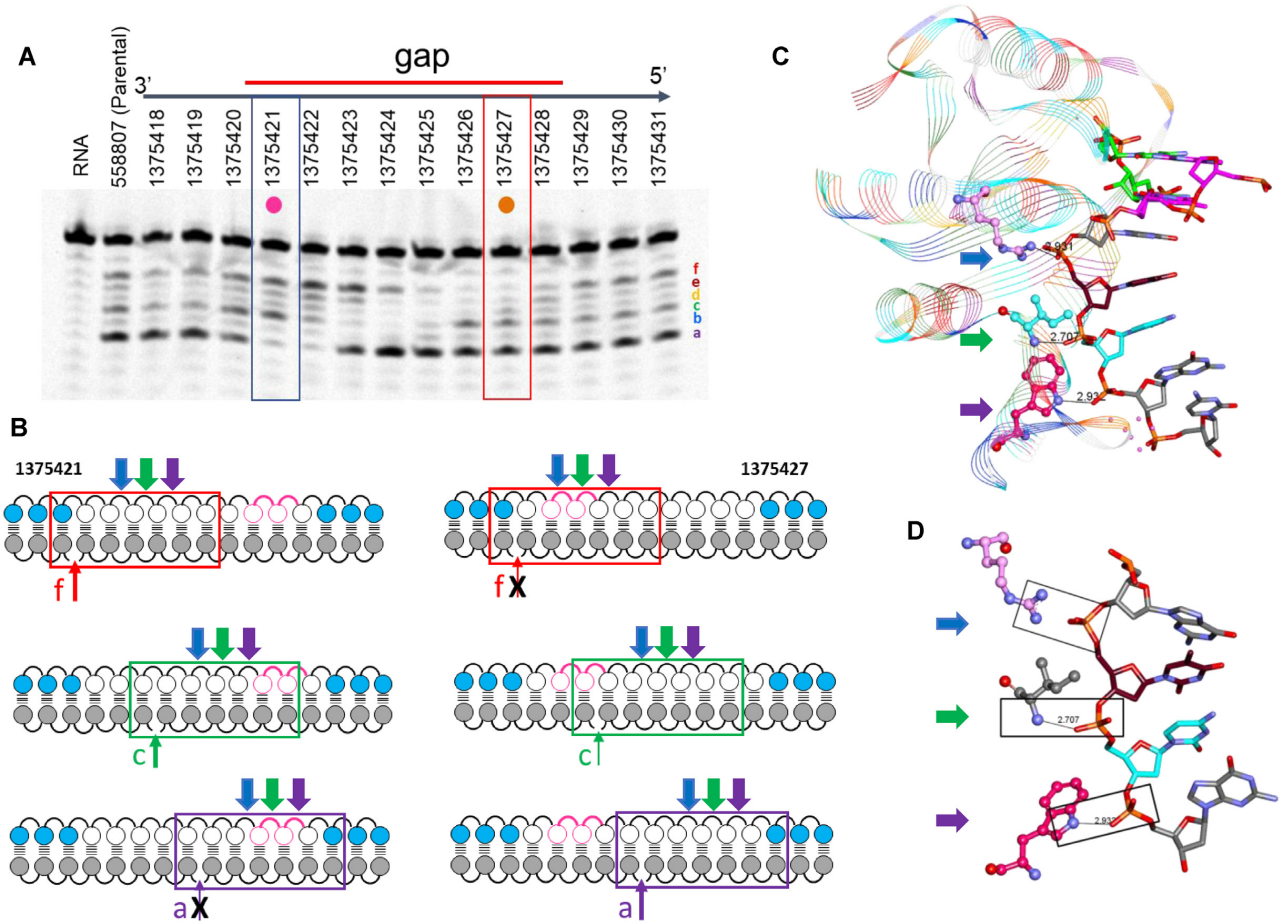
MsPA linkages have a site-specific effect on modulating RNAseH1 cleavage patterns. (**A**) Effect of introducing 2 MsPA linkages across the length of the ASO on recombinant full-length RNaseH1 cleavage patterns. (**B**) The seven-nucleotide footprint of the catalytic domain of RNaseH1 for different cleavage sites on the RNA. Pink spheres and curved lines indicates site of MsPA modification, blue spheres indicate cEt, white indicate DNA, and grey indicate RNA nucleotides. (**C**) Structural features of the seven-nucleotide footprint of the RNAseH1 catalytic domain. (**D**) Boxes show important interactions between specific amino acids and the DNA backbone. Blue arrow shows the location of the phosphate binding pocket, green shows interaction with Ile 239 and purple arrow shows interaction with Trp225.

We investigated the effect of MsPA linkages on RNaseH1 cleavage patterns on ASO/RNA heteroduplexes to determine if there were any optimal sites on the ASO for introducing MsPA linkages. ASOs where two MsPA were walked across the length of the ASO were duplexed with complementary RNA and treated with recombinant full-length human RNaseH1 (23). The enzyme produced seven cleavage sites (a–f) on the RNA strand of the heteroduplex with highest intensity at site a followed by site c and f (Figure 3A). Introducing two MsPA linkages in the 3′- or the 5′-wings had minimal effect on cleavage patterns but introducing MsPA linkages in the DNA gap had a site-specific impact on cleavage patterns.

The effect on MsPA on RNaseH1 cleavage patterns could be best rationalized by examining the two ASO sequences, 1375421 and 1375427. 1375421 causes ablation of cleavage site a. In contrast, 1375427 causes ablation of site f. The catalytic domain of RNAseH1 has a distinct but over-lapping 7 nucleotide footprint on the ASO for every cleavage site on the RNA (Figure 3B and C) (24). Introducing MsPA at positions 8, 9 in the DNA gap of 1375421 places the MsPA linkages at positions 4 and 5 of the footprint and disrupts important contacts between the ASO backbone and the amide of isoleucine 239 and tryptophan 225 (Figure 3D) (25). In contrast, MsPA at positions 8, 9 in the DNA gap is not within the footprint for cleavage site f and outside the backbone contact region for cleavage site c, allowing cleavage at this site. Similarly, MsPA at positions 2, 3 in the DNA gap of 137427 ablates important contacts in the phosphate binding pocket and the backbone amide, while they are outside the footprint of the catalytic domain for cleavage site a (Figure 3B and D).

Interestingly, uniformly modifying the gap with MsPA did not completely block cleavage but dramatically reduced the cleavage intensity for all the cleavage sites (Supplementary Figure S5). Furthermore, controlling chirality of the MsPA linkages did not alter the cleavage sites relative to the racemic MsPA linkages suggesting that chirality of MsPA linkage does not impact interactions with the catalytic domain of RNaseH1 (Supplementary Figure S6).

### Determining the number and position of PS linkages in the DNA gap that can be replaced with MsPA for maintaining activity

**Figure 4.**
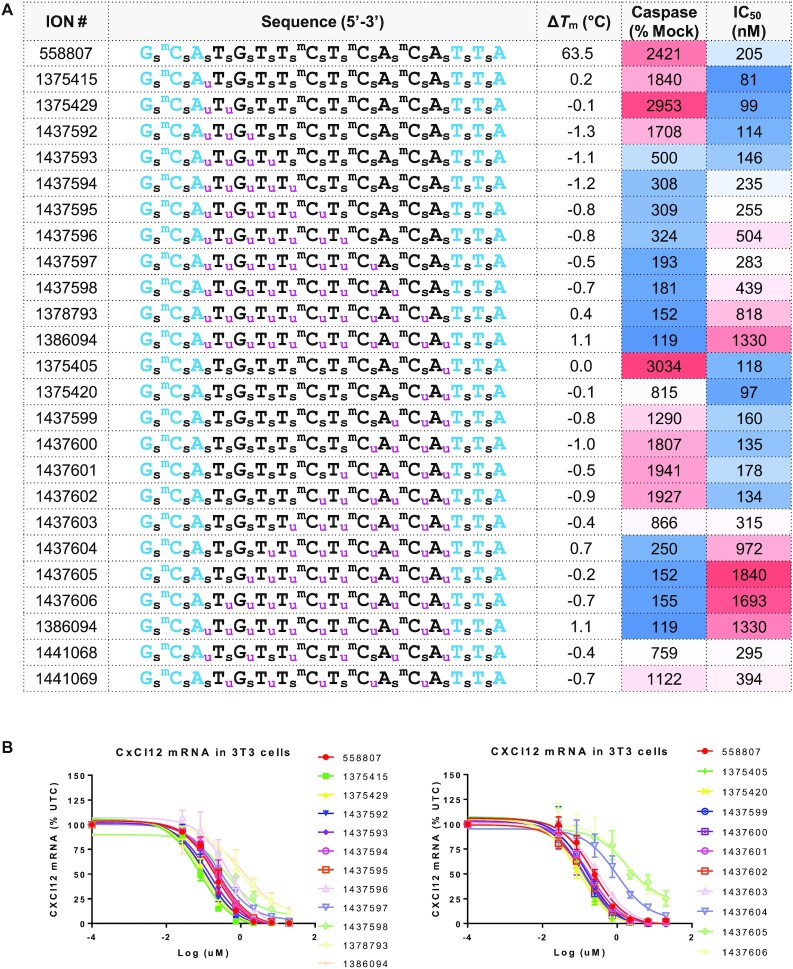
The effect of sequentially replacing PS from the 3′- or 5′-end of the gap with MsPA on antisense activity and cytotoxicity. (**A**) Design, Tm, cytotoxicity and antisense activity of ASOs with increasing MsPA linkages from the 3′ or the 5′-gap junction. (**B**) Dose–response curves for reducing CXCl12 mRNA in NIH 3T3 cells. Blue letters indicate cEt, black DNA, subscript pink u = MsPA, s = PS, superscript m = 5-Me group on cytosine nucleobases.

We next determined the optimal number of PS linkages in the DNA gap that can be replaced with MsPA while maintaining potency. We sequentially replaced 1–11 PS with MsPA starting from the 5′-gap junction or the 3′-gap junction and determined the effect of these changes on potency in cells. Replacing up to 7 PS linkages with MsPA starting from the 5′- or the 3′-gap junctions provided ASOs that were similar in potency as the parent 558807 (Figure 4A and B). Replacing nine or more PS with MsPA from either side of the gap resulted in significant reduction in potency. We also alternated PS and MsPA linkages in two registers and found that ASOs with both designs showed similar potency as the parent ASO.

In contrast to the effects on potency, the directionality of replacing PS with MsPA in the gap had a remarkable effect on cytotoxicity. As observed in our previous studies with 2′- and 5′-modifications (7,8), replacing PS in the vicinity of the 5′-cEt/gap junction reduced cytotoxicity whereas similar substitution near the 3′-cEt/DNA gap junction did not have a strong effect for reducing cytotoxicity.

### Determining the number and position of PS linkages in the DNA gap that can be replaced with MsPA while retaining potency and enhancing therapeutic index in mice

**Figure 5.**
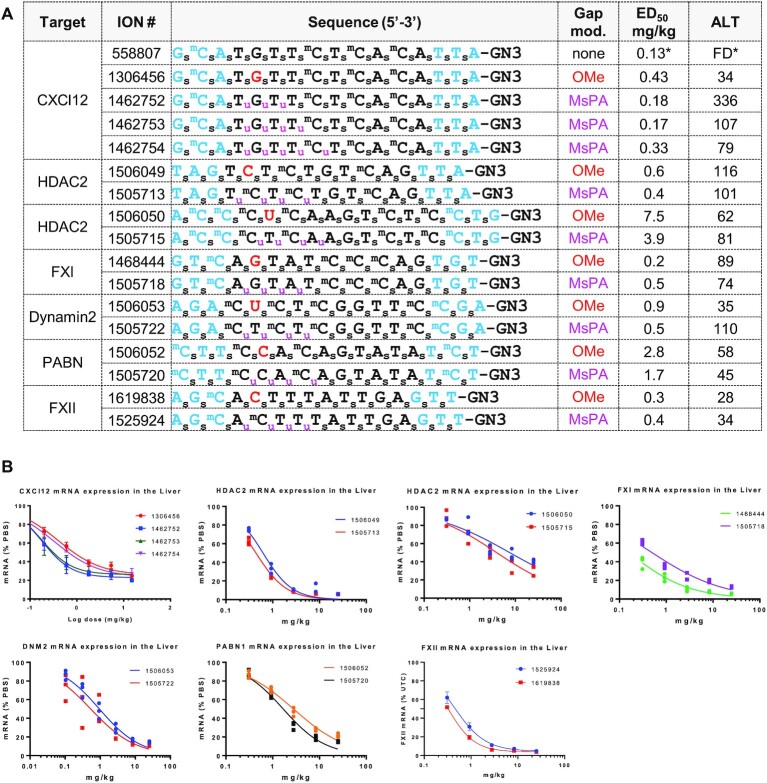
MsPA ASOs show improved therapeutic index in mice. (**A**) Gene target, sequence, design of MsPA ASOs, potency for reducing targeted mRNA and plasma transaminase levels following ASO administration. Mice Balb-c (*n* = 3/group) were subcutaneously injected with 0.2, 0.6, 1.8, 5.4 and 15 mg/kg of CXCl12 ASOs or 0.3, 0.9, 2.8, 8.3 and 25 mg/kg of HDAC2, FXI, Dynamin 2, PABN, and FXII ASOs and the animals were sacrificed 72 h after injection. Livers were harvested and reduction of target mRNA in the liver was quantified by qRT-PCR, and plasma ALT levels were measured post-sacrifice. FD = found dead, *indicates data from a different experiment. (**B**) Dose response curves for reducing the targeted mRNA in mice livers. Blue letters indicate cEt, black DNA, red – 2′-*O*-Me, GN3 = trivalent THA-GalNAc conjugate, subscript pink u = MsPA, s = PS, superscript m = 5-Me group on cytosine nucleobases.

We determined the optimal number of MsPA near the 5′-cEt/gap junction to reduce the hepatotoxicity of some model toxic cEt gapmer PS ASOs in mice. We first examined the effect of replacing 3, 4 and 5 PS with MsPA at DNA gap positions 1–5 on mitigating hepatotoxicity of parent ASO 558807 (Figure 5A and B). The ASOs were synthesized as GalNAc conjugates which enhances ASO potency by increasing ASO delivery to hepatocytes (26). We also evaluated the ASO with 2′-OMe (OMe) at position 2 in the gap (1306456) as a control that we showed previously can dramatically reduce hepatotoxicity (7). Mice (Balbc, *n* = 4/group) were injected subcutaneously with 0.2, 0.6, 1.8, 5.4 and 15 mg/kg of ASO and the animals were sacrificed 72 hours after injection. Livers were harvested and reduction of CXCl12 mRNA in the liver was quantified by qRT-PCR, and plasma ALT levels were measured post-sacrifice. All the MsPA ASOs showed similar or improved potency as compared with the OMe control ASOs. The MsPA ASOs also significantly improved hepatotoxicity profile relative to the parent ASO, which is very toxic when administered at 15mg/kg as a GalNAc-conjugate. The ASO with 3 MsPA in the gap (1462752) showed modest ALT elevations at the highest dose of 15 mg/kg while ASOs with 4 and 5 MsPA in the gap (1462753, 1462754) were safe at this dose.

The ASO with 4 MsPA in the gap (1462753) showed the optimal potency and mitigation of cyto- and hepatotoxicity in the experiment above and as result this design was chosen for evaluation of additional model toxic ASOs targeting HDAC2, FXI, PABN, Dynamin 2 mRNAs (Figure 5A and B). All the parent ASOs were previously shown to be hepatotoxic in mice (8,10) and were not evaluated in these studies to reduce animal usage. Instead, we used ASOs with OMe at position 2 in the gap as controls to determine if MsPA substitution could maintain potency and safety relative to this design. Mice (Balbc, *n* = 4/group) were injected subcutaneously with 0.3, 0.9, 2.8, 8.3 and 25 mg/kg of the ASOs and the animals were sacrificed 72 h after injection. All the MsPA and OMe ASOs mitigated the hepatoxicity caused by their respective parent ASOs at the highest dose of 25 mg/kg administered as the respective GalNAc-ASO conjugates (corresponds to 250 mg/kg of unconjugated ASO). In general, the MsPA ASOs were ∼2-fold more potent than their OMe counterparts based on the effective dose to reduce the targeted mRNA by 50% (ED_50_) in the liver.

### MsPA reduces non-specific protein binding of PS ASOs

**Figure 6.**
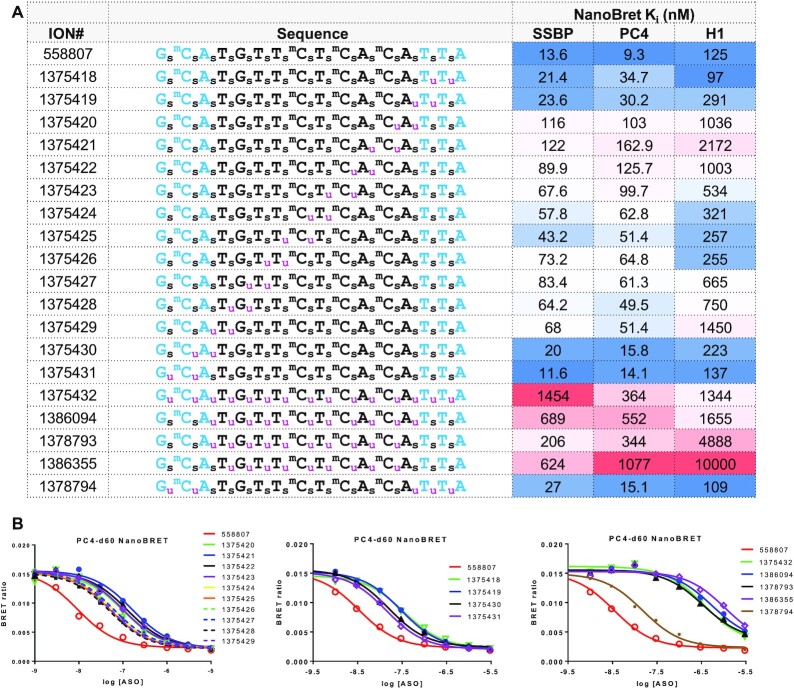
MsPA modulates protein-binding profile of gapmer ASOs. (**A**) Competition binding constants of MsPA ASOs for model PS ASO binding proteins, PC4, SSBP and RNaseH1 using a NanoBret assay. (**B**) Dose response curves showing that MsPA in the DNA gap has a bigger effect on protein binding than MsPA in the cEt wings. Blue letters indicate cEt, black DNA, red—2′-*O*-Me, subscript pink m = MsPA, s = PS, superscript m = 5-Me group on cytosine nucleobases.

We next determined the effect of the MsPA modification on modulating the protein binding properties of PS ASOs. MsPA ASOs described in Figure 1 where the entire ASO, or only the gap, or the wings of the ASO were MsPA-modified and where two MsPA were walked across the entire length of the ASO, were evaluated using a NanoBRET assay (17) versus three model proteins—PC4, single strand binding protein (SSBP) and RNaseH1. Interaction of PS ASOs with these model proteins can be used to better understand the effect of chemical modifications on modulating ASO-protein interactions (18). Replacing all PS in the ASO or only PS in the gap with MsPA (1375432, 1386094, 1378793 and 1386355) had a dramatic effect at reducing non-specific protein binding (Figure 6A-B and Supplementary Figure S7). Interestingly, replacing two MsPA in either the 5′- or the 3′-cEt wings (1375418, 1375431 and 1378794) had a minimal effect whereas replacing two MsPA in the DNA gap (1375420–1375429) had a much stronger effect for mitigating protein binding. DNA nucleotides lack a 2′-group that sterically impedes access of amino acid side-chains to the sulfur atom in the PS linkage to establish polar/hydrophobic interactions that drive the interactions of PS ASOs with proteins (5). This reduced protein binding was also accompanied with reduced hepatotoxicity and reduced elevation of P21 mRNA levels in mouse liver (Supplementary Figure S8A), and with reduced nucleolar mislocalization of P54nrb in cells (Supplementary Figure S8B), suggesting that MsPA modification reduces apoptotic cell death, consistent with the mechanism of toxicity determined previously (7).

### MsPA reduces the pro-inflammatory profile of PS ASOs

**Figure 7.**
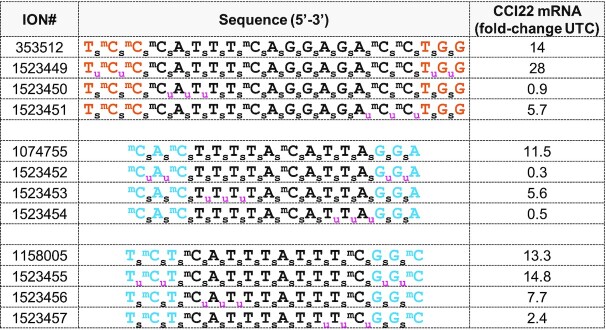
MsPA reduces cytokine induction observed with model pro-inflammatory gapmer ASOs in BJAB cells. Sequence and design of ASOs and fold increase in CCl22 mRNA in BJAB cells as measured by qRT-PCR, following treatment with 1.6 mM ASO by free-uptake for 16 h. Blue letters indicate cEt, black DNA, red—2′-*O*-Me, subscript pink u = MsPA, s = PS, superscript m = 5-Me group on cytosine nucleobases.

PS ASOs can cause pro-inflammatory effects in cell and animal models that is PS and sequence dependent (27). Those proinflammatory effects are mostly modulated in a TLR9 dependent manner even in absence of canonical CpG motif resulting in release of cytokines and immune cell infiltration. We used BJAB cells, an EBV-negative Burkitt-like lymphoma cell line, which is uniquely sensitive to stimulation by CpG and non-CpG oligonucleotides in a sequence and TLR9 dependent fashion. We treated BJAB cells using model pro-inflammatory ASOs 353512, 1074755 and 1158005 to determine the effect of MsPA linkages on cytokine (CCl22) induction as measured by qRT-PCR (Figure 7). We replaced two PS on either ends of the ASO with MsPA, or replaced 3 PS on the 3′- or the 5′-end of the DNA gap with MsPA. These designs were employed as we were not certain *a priori* which regions of the PS ASOs were responsible for interaction with immune cell proteins/receptors.

Interestingly, the effect of MPA modifications on CCl22 induction was different for the ASO sequences evaluated. For the 3–14–3 MOE gapmer 353512, replacing PS with MsPA at the 5′-gap junction was most effective at mitigating CCl22 induction. In contrast, MsPA at the 3′-gap junction was more effective for the 3–10–3 cEt gapmer ASOs 1074755 and 1158005, respectively. These data suggest that different regions of the ASO interact with immune receptors and replacing PS with MsPA at these locations can interfere with these interactions to reduce pro-inflammatory effects.

We also evaluated the MsPA modified CXCl12 ASOs for cytokine induction, even though the parent ASO 558807 is not pro-inflammatory and produces only modest changes in CCl22 in BJAB cells, to determine if MsPA can enhance the pro-inflammatory profile of an otherwise non pro-inflammatory ASO (Supplementary Figure S9). All the MsPA ASOs mitigated the modest CCl22 mRNA inductions produced by 558807 suggesting that the MsPA linkage does not increase pro-inflammatory effects relative to PS. Presumably, mitigating non-specific protein binding of PS ASOs using MsPA reduces interactions with proteins/receptors on immune cells resulting in a reduced pro-inflammatory profile.

### MsPA increase the duration of effect of PS ASOs

Given the improved nuclease stability observed with MsPA linkages, we determined if replacing PS with MsPA in the DNA gap could enhance the duration of pharmacodynamic effects of gapmer ASOs in mice. We evaluated MsPA modified 3–10–3 cEt gapmer ASOs targeting mouse FXII as this protein is synthesized in hepatocytes and secreted in the blood thus serving as a marker for antisense activity in the liver over time. Since the cEt modification greatly enhances nuclease stability (20), we only examined the effect of introducing MsPA at the cEt/DNA gap junctions or in the DNA gap on duration of FXII protein knockdown in plasma. We also included ASOs where the PS linkages in the cEt wings were replaced with PO, and a chemically stabilized siRNA versus FXII for comparison (3). Mice were injected with a single dose of the ASOs at a dose that produced 80% FXII protein knockdown in plasma (0.9 mg/kg) and recovery of FXII protein was measured at 2, 4, 7, 14, 21, 28 and 35 days post injection. In general, we observed a slightly longer duration of effect for the ASO where four PS in the DNA gap were replaced with MsPA, while replacing PS with PO in the cEt wings had a minimal effect on duration (Supplementary Figure S10). These data are consistent with previous observations that gapmer ASOs are metabolized by endo-nucleolytic cleavage in the DNA gap whereas the 2′-modified wings are more stable and not prone to nucleolytic degradation (28).

**Figure 8.**
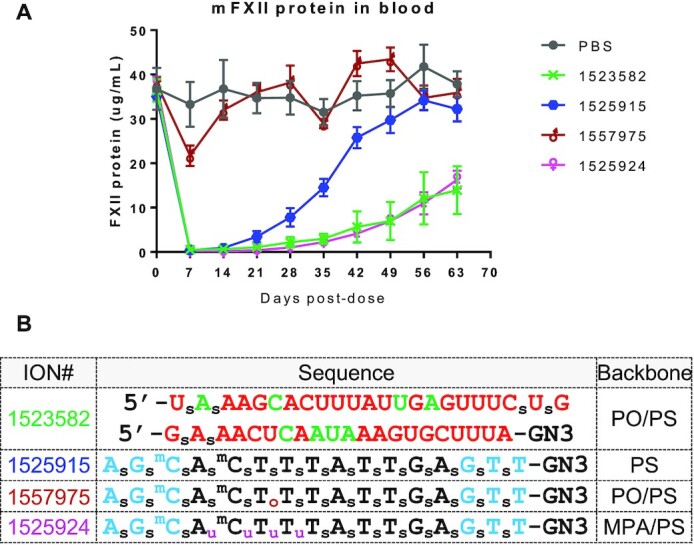
MsPA increases ASO duration of effect in mice. (**A**) Recovery of FXII protein in mouse plasma following subcutaneous injection of ASOs or siRNA. ASOs and siRNA were injected subcutaneously at 10-fold the ED_80_—8 mg/kg for the ASOs and 5 mg/kg for the siRNA. (**B**) Sequence and design of siRNA and ASOs used for the experiment. Blue letters indicate cEt, black DNA, red—2′-*O*-Me, green 2′F, GN3 indicates triantennary THA-GalNac conjugate, subscript pink u = MsPA, s = PS, o = PO, superscript m = 5-Me group on cytosine nucleobases.

To determine if the effect on duration could be amplified by using a higher dose, we injected the parent ASO 1525915, ASO 1525924 where four PS in the gap were replaced with MsPA, ASO 1557975 which had a metabolically labile PO linkage in the gap and the FXII siRNA 1523582 in a follow-up experiment (Figure 8). All ASOs except, 1557975 (PO in gap), showed complete knockdown of FXII protein in the blood until the week 3 time point. FXII protein levels started to recover and were back to baseline by week 8 for the parent gapmer 1525915. In contrast, the MsPA ASO and the stabilized siRNA showed close to 60% suppression of plasma FXII protein even at the 8-week timepoint. The siRNA design used in our experiment is similar to the designs that show extended pharmacodynamic half-lives in clinical trials (29) suggesting that MsPA ASOs have the potential for extending the duration of effect of gapmer ASOs in the clinic and allow for quarterly or bi-annual dosing regimens.

## DISCUSSION

The PS modification and DNA gapmers represent the most widely investigated oligonucleotide modification and ASO design in the clinic respectively (6). At least five PS ASOs have been approved by regulatory agencies and over 40 gapmer ASOs are currently in pre-clinical or clinical development for a variety of disease indications (30). PS ASOs can be delivered by subcutaneous, intravenous intrathecal, intravitreal, aerosol and oral routes of administration depending on the tissues being targeted for treatment (11). Upon systemic administration, PS ASOs distribute broadly but accumulate preferentially in the liver and the kidneys.

The PS modification is indispensable for gapmer therapeutics as it stabilizes the single stranded DNA gap from nucleolytic degradation and is one of very few backbone modifications that supports RNaseH1-mediated degradation of RNA (2). PS also enhances ASO interactions with proteins. PS-mediated protein binding promotes ASO interactions with plasma proteins to facilitate distribution from the injection site to tissues, with cell-surface proteins to promote entry into cells (31), and with intra-cellular proteins to modulate activity and toxicity (4). Plasma proteins that bind PS ASOs have been characterized (32) and several cell-surface proteins and intra-cellular proteins that interact with PS ASOs have also been identified (33,34).

The PS ASO design obviates the need for complex nanoparticles or delivery agents and these agents can be easily administered by subcutaneous injections in saline. Indeed, the use of chemically stabilized oligonucleotides, with or without conjugates and/or targeting moieties, represents the cornerstone of modern oligonucleotide delivery strategies. However, the non-specific protein binding of PS ASOs, if too tight, can be a double-edged sword resulting in pro-inflammatory effects leading to undesirable clinical outcomes. Therefore, chemical strategies that can modulate protein binding of ASOs while maintaining antisense activity and chemical stability could be broadly beneficial.

We recently showed that small structural changes in the ASO such as site-specific introduction of 2′- and 5′-substitutions on the 2′-deoxynucleotide sugar (7,8), replacing one or two PS with alkyl-phosphonate linkages (10), or replacing the natural 3′,5′-PS linkage with a 2′,5′-linkage (9) could mitigate ASO toxicities without compromising activity. However, these ASO designs still retained significant protein binding as they did not reduce the total number of PS linkages in the ASO, a major determinant of protein binding. In this work, we evaluated the effect of replacing PS with MsPA linkages to investigate the effect of this modification on ASO activity and protein binding properties.

The MsPA modifcation was recently reported by Stetsenko as an alternate for PS in uniform DNA ASOs targeting miR-21 (13). Uniform DNA MsPA oligonucleotides administered in complex with folate-containing liposomes demonstrated RNaseH1-mediated inhibition of primary tumor growth by downregulating miR-21 in tumors and increased biosynthesis of miR-21–regulated tumor suppressor proteins (12). Peritumoral administration of MsPA DNA ASOs resulted in efficient accumulation in the tumor. Stetsenko also reported that a MsPA modified uniform MOE ASO showed similar activity as nusinersen (full PS MOE ASO) in cells following delivery by transfection but significantly reduced activity in mice (14). Presumably, the reduced protein binding of full MsPA ASOs adversely affected tissue distribution and cellular uptake. However, the effect of incorporating MsPA in state-of-the-art gapmer ASOs with high affinity modifications such as MOE and cEt (2), and the effect of combining PS with MsPA to retain sufficient protein binding to facilitate tissue distribution, cell uptake and endosomal release were not investigated.

The MsPA linkage was introduced into oligonucleotides during the oxidation cycle by a Staudinger reaction between mesylazide and the phosphite intermediate formed after phosphoramidite coupling (19). As a result, MsPA can be inserted at any location in an oligonucleotide and combined with other backbone and sugar-modifications. Unlike alkyl-phosphonates and phosphoramidate backbones, MsPA oligonucleotides show high chemical stability and are stable to acid required for the removal of DMTr-protecting groups during oligonucleotide synthesis and to aqueous ammonia for deprotection of the oligonucleotide following completion of synthesis. MsPA ASOs showed similar RNA-affinity as PS ASOs and can be seamlessly combined with sugar modifications without adversely impacting the melting temperature of ASO/RNA duplexes.

Gapmer ASOs uniformly modified with MsPA across the entire ASO or just in the DNA gap, were active but showed reduced potency and efficacy. This was contrary to earlier reports where uniform DNA MsPA ASOs were reported to have improved RNase H1 activation relative to PS DNA ASOs (13). It is conceivable that uniform DNA provides a wider window for RNase H1 activity which is more important for fully-modified MsPA ASOs. However, we observed reduced potency and efficacy with full MsPA modification in the context of gapmer ASOs which have a narrower region of ∼10 nucleotides for RNase H1 cleavage. Instead, replacing up to 5 PS in the gap with MsPA showed similar or improved activity, demonstrating wide tolerance of the MsPA substitution in the gap, provided some DNA PS is retained.

Replacing two or more PS near the 5′-side of the gap with MsPA reduced cytotoxicity and hepatotoxicity of a very toxic model gapmer ASO targeting CXCl12 mRNA, consistent with our previous observations that the 5′-side of the gap is sensitive to modulate cytotoxicity. Interestingly, controlling the chirality of the MsPA linkages did not provide any discernible advantage for enhancing potency or nuclease stability or for reducing cytotoxicity. MsPA ASOs generally supported RNAseH1-mediated cleavage of complementary RNA but uniformly modified designs showed reduced cleavage intensity. Site-specific incorporation of two MsPA in the gap produced changes in cleavage patterns although the effect on overall cleavage rates was not evaluated. In general, the data suggest that MsPA is not well tolerated in the phosphate binding pocket and at two adjacent sites where the catalytic domain of RNAseH1 makes intimate contacts with the ASO backbone.

ASO designs with 3, 4 and 5 MsPA linkages at positions 1–5 on the 5′-side of the DNA gap were evaluated in mice using a model toxic ASO targeting CXCl12 mRNA. The ASO with 4 MsPA at gap positions 1–4 showed optimal potency and mitigation of hepatotoxicity. This design was further evaluated using additional ASO sequences targeting HDAC2, FXI, PABN, Dynamin2 and FXII mRNA and compared to ASOs with OMe at gap position 2. In all cases, the MsPA ASOs showed similar or slightly improved potency as compared to the OMe ASOs and broadly mitigated hepatotoxicity suggesting that 3–5 MPA at the 5′-end of the DNA gap could be a broadly useful design to improve therapeutic index.

The MsPA ASOs were also evaluated in a NanoBRET assay using model proteins to determine the effect of MsPA incorporation on protein binding of PS ASOs (17). Replacing all PS with MsPA or replacing PS in the DNA gap with MsPA had a dramatic effect on reducing protein binding. Replacing PS with MsPA in the cEt wings had a modest or no effect on protein binding. In contrast, replacing two PS with MsPA in the gap had a significant impact on reducing protein binding. To determine if these effects were biologically meaningful, we replaced 3–4 PS with MsPA at different locations of three model pro-inflammatory ASOs and measured the effect of these changes on CCl22 induction in BJAB cells. Interestingly, replacing PS with MsPA near the 5′-side of the gap was effective at mitigating the pro-inflammatory profile of one ASO while substitution at the 3′-side of the gap was more effective for two other ASOs. These observations suggest that ASO interactions with proteins of the immune system may be affected by the chemical modification and that replacing PS with MsPA can be a general chemical strategy to mitigate these interactions and reduce pro-inflammatory effects.

The MsPA linkage also enhanced stability from nuclease digestion relative to PS. Since the PS DNA gap is the metabolic weak link in gapmer ASOs (28), we evaluated the effect of replacing PS with MsPA on duration of ASO activity in mice. The study was carried out using an ASO targeting mouse FXII as this protein is synthesized in hepatocytes and secreted in the plasma and serves as a bio-marker for antisense effects in the liver. Replacing PS with MsPA in the cEt wings had minimal impact on duration, likely because the cEt modification provides significant nuclease stability (35). In contrast, replacing 4 PS with MsPA in the gap had a profound impact on ASO duration which was now comparable to the duration of an enhanced stabilized siRNA (3). This result suggests that MsPA modification in the DNA gap could be a general strategy for enhancing ASO duration of effect in the clinic and allow for quarterly or bi-annual dosing regimens.

In conclusion, we demonstrate that site-specific replacement of PS linkages with MsPA can enhance the potency and therapeutic index of gapmer ASOs without compromising antisense activity and chemical stability – crucial attributes for developing the next generation of ASO therapeutics. MsPA ASOs showed reduced non-specific protein binding, decreased cyto- and hepato-toxicity, and reduced pro-inflammatory effects. The improved nuclease stability of MsPA over PS also translated to significant improvement in the duration of ASO action in mice. Given these attributes, MsPA/PS ASOs represent the next generation of gapmer ASOs with the ability to modulate ASO protein binding, therapeutic index and duration of effect.

## Supplementary Material

gkab718_Supplemental_File
