# Supplementary material for: Towards next generation antisense oligonucleotides: mesylphosphoramidate modification improves therapeutic index and duration of effect of gapmer antisense oligonucleotides
Source: Nucleic Acids Res. 2021 Aug 20;49(16):9026–41. doi: 10.1093/nar/gkab718 (PMC8450106; doi:10.1093/nar/gkab718)
Supplement: gkab718_Supplemental_File [file gkab718_supplemental_file.pptx]

## Slide 1
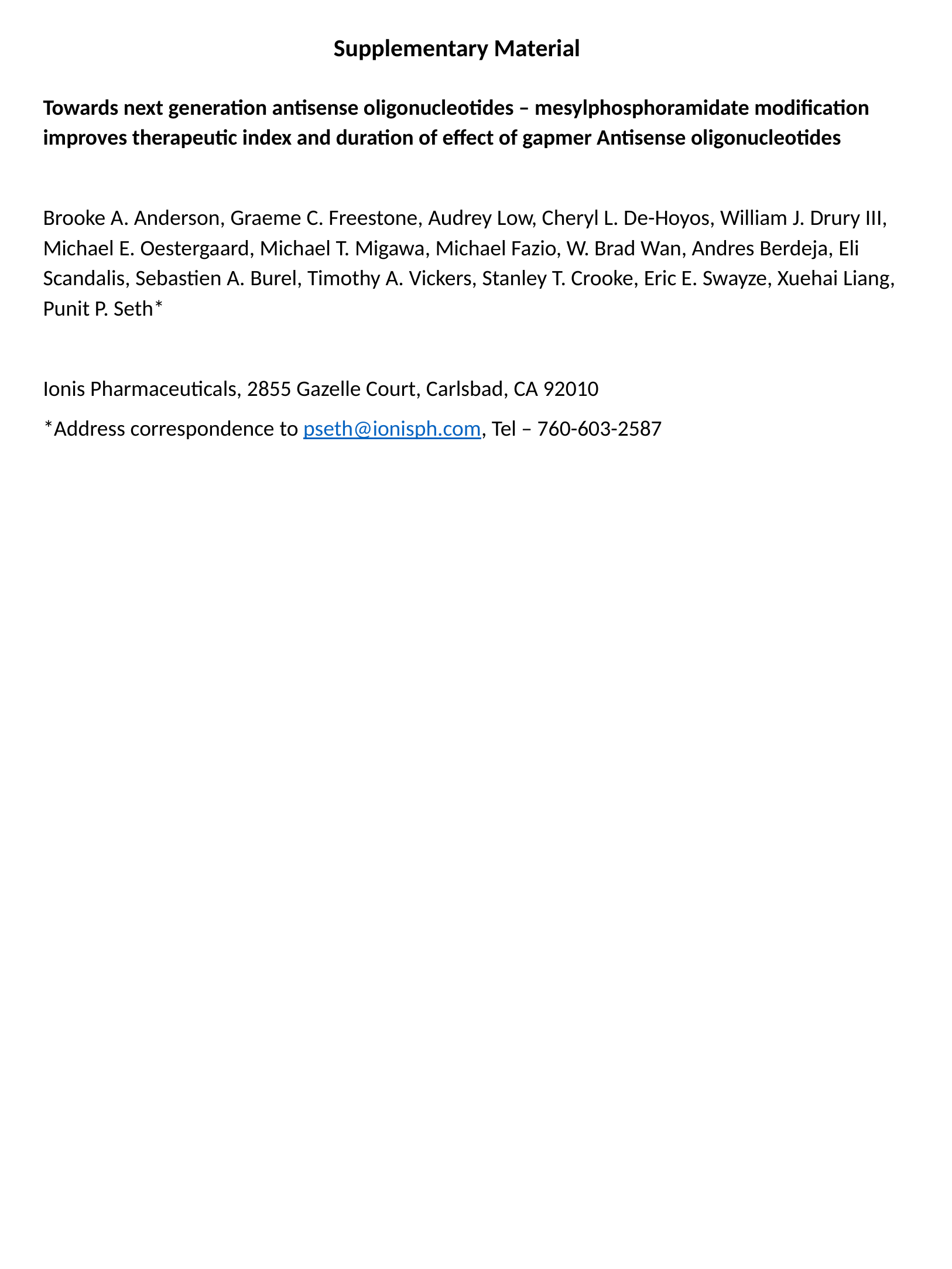

Supplementary Material
Towards next generation antisense oligonucleotides – mesylphosphoramidate modification improves therapeutic index and duration of effect of gapmer Antisense oligonucleotides
Brooke A. Anderson, Graeme C. Freestone, Audrey Low, Cheryl L. De-Hoyos, William J. Drury III, Michael E. Oestergaard, Michael T. Migawa, Michael Fazio, W. Brad Wan, Andres Berdeja, Eli Scandalis, Sebastien A. Burel, Timothy A. Vickers, Stanley T. Crooke, Eric E. Swayze, Xuehai Liang, Punit P. Seth*
Ionis Pharmaceuticals, 2855 Gazelle Court, Carlsbad, CA 92010
*Address correspondence to pseth@ionisph.com, Tel – 760-603-2587

## Slide 2
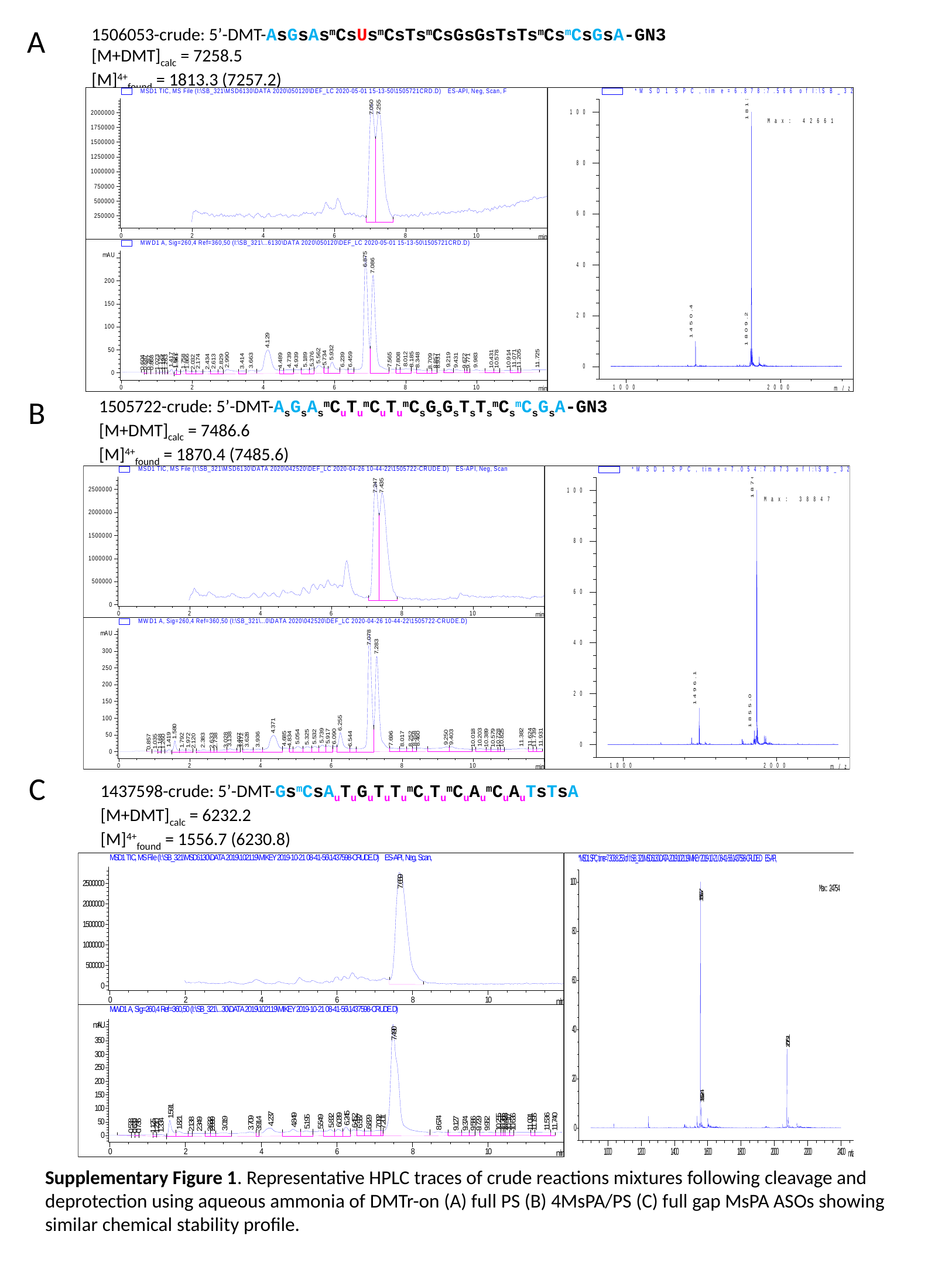

A
1506053-crude: 5’-DMT-AsGsAsmCsUsmCsTsmCsGsGsTsTsmCsmCsGsA-GN3
[M+DMT]calc = 7258.5
[M]4+found = 1813.3 (7257.2)
B
1505722-crude: 5’-DMT-AsGsAsmCuTumCuTumCsGsGsTsTsmCsmCsGsA-GN3
[M+DMT]calc = 7486.6
[M]4+found = 1870.4 (7485.6)
C
1437598-crude: 5’-DMT-GsmCsAuTuGuTuTumCuTumCuAumCuAuTsTsA​
[M+DMT]calc = 6232.2
[M]4+found = 1556.7 (6230.8)
Supplementary Figure 1. Representative HPLC traces of crude reactions mixtures following cleavage and deprotection using aqueous ammonia of DMTr-on (A) full PS (B) 4MsPA/PS (C) full gap MsPA ASOs showing similar chemical stability profile.

## Slide 3
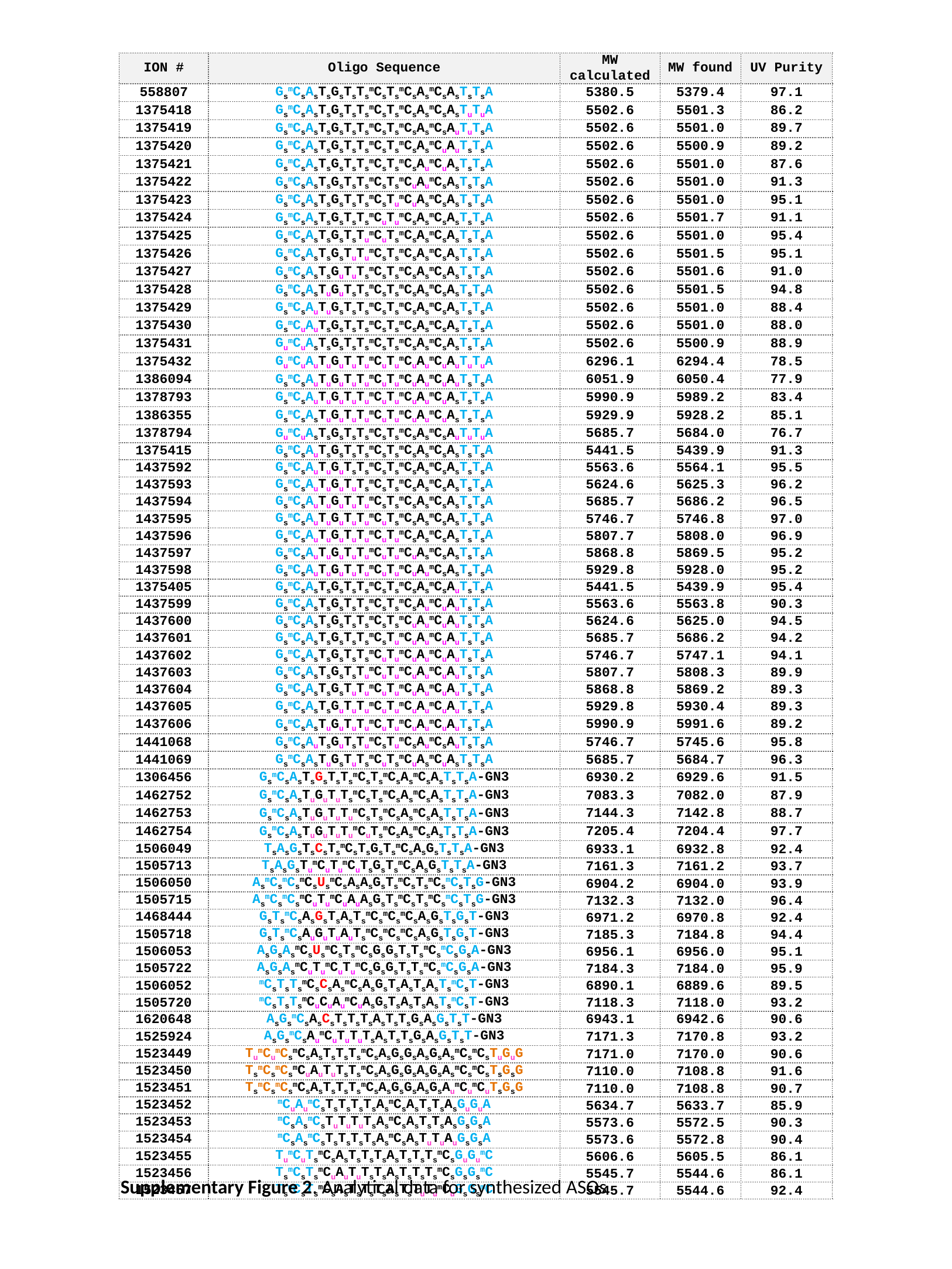

| ION #​ | Oligo Sequence​ | MW calculated | MW found | UV Purity |
| --- | --- | --- | --- | --- |
| 558807​ | GsmCsAsTsGsTsTsmCsTsmCsAsmCsAsTsTsA | 5380.5 | 5379.4 | 97.1 |
| 1375418​ | GsmCsAsTsGsTsTsmCsTsmCsAsmCsAsTuTuA | 5502.6 | 5501.3 | 86.2 |
| 1375419​ | GsmCsAsTsGsTsTsmCsTsmCsAsmCsAuTuTsA | 5502.6 | 5501.0 | 89.7 |
| 1375420​ | GsmCsAsTsGsTsTsmCsTsmCsAsmCuAuTsTsA | 5502.6 | 5500.9 | 89.2 |
| 1375421​ | GsmCsAsTsGsTsTsmCsTsmCsAumCuAsTsTsA | 5502.6 | 5501.0 | 87.6 |
| 1375422​ | GsmCsAsTsGsTsTsmCsTsmCuAumCsAsTsTsA | 5502.6 | 5501.0 | 91.3 |
| 1375423​ | GsmCsAsTsGsTsTsmCsTumCuAsmCsAsTsTsA | 5502.6 | 5501.0 | 95.1 |
| 1375424​ | GsmCsAsTsGsTsTsmCuTumCsAsmCsAsTsTsA | 5502.6 | 5501.7 | 91.1 |
| 1375425​ | GsmCsAsTsGsTsTumCuTsmCsAsmCsAsTsTsA | 5502.6 | 5501.0 | 95.4 |
| 1375426​ | GsmCsAsTsGsTuTumCsTsmCsAsmCsAsTsTsA | 5502.6 | 5501.5 | 95.1 |
| 1375427​ | GsmCsAsTsGuTuTsmCsTsmCsAsmCsAsTsTsA | 5502.6 | 5501.6 | 91.0 |
| 1375428​ | GsmCsAsTuGuTsTsmCsTsmCsAsmCsAsTsTsA | 5502.6 | 5501.5 | 94.8 |
| 1375429​ | GsmCsAuTuGsTsTsmCsTsmCsAsmCsAsTsTsA | 5502.6 | 5501.0 | 88.4 |
| 1375430​ | GsmCuAuTsGsTsTsmCsTsmCsAsmCsAsTsTsA | 5502.6 | 5501.0 | 88.0 |
| 1375431​ | GumCuAsTsGsTsTsmCsTsmCsAsmCsAsTsTsA | 5502.6 | 5500.9 | 88.9 |
| 1375432​ | GumCuAuTuGuTuTumCuTumCuAumCuAuTuTuA | 6296.1 | 6294.4 | 78.5 |
| 1386094​ | GsmCsAuTuGuTuTumCuTumCuAumCuAuTsTsA | 6051.9 | 6050.4 | 77.9 |
| 1378793​ | GsmCsAuTuGuTuTumCuTumCuAumCuAsTsTsA | 5990.9 | 5989.2 | 83.4 |
| 1386355​ | GsmCsAsTuGuTuTumCuTumCuAumCuAsTsTsA | 5929.9 | 5928.2 | 85.1 |
| 1378794​ | GumCuAsTsGsTsTsmCsTsmCsAsmCsAuTuTuA | 5685.7 | 5684.0 | 76.7 |
| 1375415​ | GsmCsAuTsGsTsTsmCsTsmCsAsmCsAsTsTsA | 5441.5 | 5439.9 | 91.3 |
| 1437592​ | GsmCsAuTuGuTsTsmCsTsmCsAsmCsAsTsTsA | 5563.6 | 5564.1 | 95.5 |
| 1437593​ | GsmCsAuTuGuTuTsmCsTsmCsAsmCsAsTsTsA | 5624.6 | 5625.3 | 96.2 |
| 1437594​ | GsmCsAuTuGuTuTumCsTsmCsAsmCsAsTsTsA | 5685.7 | 5686.2 | 96.5 |
| 1437595​ | GsmCsAuTuGuTuTumCuTsmCsAsmCsAsTsTsA | 5746.7 | 5746.8 | 97.0 |
| 1437596​ | GsmCsAuTuGuTuTumCuTumCsAsmCsAsTsTsA | 5807.7 | 5808.0 | 96.9 |
| 1437597​ | GsmCsAuTuGuTuTumCuTumCuAsmCsAsTsTsA | 5868.8 | 5869.5 | 95.2 |
| 1437598​ | GsmCsAuTuGuTuTumCuTumCuAumCsAsTsTsA | 5929.8 | 5928.0 | 95.2 |
| 1375405​ | GsmCsAsTsGsTsTsmCsTsmCsAsmCsAuTsTsA | 5441.5 | 5439.9 | 95.4 |
| 1437599​ | GsmCsAsTsGsTsTsmCsTsmCsAumCuAuTsTsA | 5563.6 | 5563.8 | 90.3 |
| 1437600​ | GsmCsAsTsGsTsTsmCsTsmCuAumCuAuTsTsA | 5624.6 | 5625.0 | 94.5 |
| 1437601​ | GsmCsAsTsGsTsTsmCsTumCuAumCuAuTsTsA | 5685.7 | 5686.2 | 94.2 |
| 1437602​ | GsmCsAsTsGsTsTsmCuTumCuAumCuAuTsTsA | 5746.7 | 5747.1 | 94.1 |
| 1437603​ | GsmCsAsTsGsTsTumCuTumCuAumCuAuTsTsA | 5807.7 | 5808.3 | 89.9 |
| 1437604​ | GsmCsAsTsGsTuTumCuTumCuAumCuAuTsTsA | 5868.8 | 5869.2 | 89.3 |
| 1437605​ | GsmCsAsTsGuTuTumCuTumCuAumCuAuTsTsA | 5929.8 | 5930.4 | 89.3 |
| 1437606​ | GsmCsAsTuGuTuTumCuTumCuAumCuAuTsTsA | 5990.9 | 5991.6 | 89.2 |
| 1441068​ | GsmCsAuTsGuTsTumCsTumCsAumCsAuTsTsA | 5746.7 | 5745.6 | 95.8 |
| 1441069​ | GsmCsAsTuGsTuTsmCuTsmCuAsmCuAsTsTsA | 5685.7 | 5684.7 | 96.3 |
| 1306456​ | GsmCsAsTsGsTsTsmCsTsmCsAsmCsAsTsTsA-GN3 | 6930.2 | 6929.6 | 91.5 |
| 1462752​ | GsmCsAsTuGuTuTsmCsTsmCsAsmCsAsTsTsA-GN3 | 7083.3 | 7082.0 | 87.9 |
| 1462753​ | GsmCsAsTuGuTuTumCsTsmCsAsmCsAsTsTsA-GN3 | 7144.3 | 7142.8 | 88.7 |
| 1462754​ | GsmCsAsTuGuTuTumCuTsmCsAsmCsAsTsTsA-GN3 | 7205.4 | 7204.4 | 97.7 |
| 1506049 | TsAsGsTsCsTsmCsTsGsTsmCsAsGsTsTsA-GN3 | 6933.1 | 6932.8 | 92.4 |
| 1505713 | TsAsGsTumCuTumCuTsGsTsmCsAsGsTsTsA-GN3 | 7161.3 | 7161.2 | 93.7 |
| 1506050 | AsmCsmCsmCsUsmCsAsAsGsTsmCsTsmCsmCsTsG-GN3 | 6904.2 | 6904.0 | 93.9 |
| 1505715 | AsmCsmCsmCuTumCuAuAsGsTsmCsTsmCsmCsTsG-GN3 | 7132.3 | 7132.0 | 96.4 |
| 1468444 | GsTsmCsAsGsTsAsTsmCsmCsmCsAsGsTsGsT-GN3 | 6971.2 | 6970.8 | 92.4 |
| 1505718 | GsTsmCsAuGuTuAuTsmCsmCsmCsAsGsTsGsT-GN3 | 7185.3 | 7184.8 | 94.4 |
| 1506053 | AsGsAsmCsUsmCsTsmCsGsGsTsTsmCsmCsGsA-GN3 | 6956.1 | 6956.0 | 95.1 |
| 1505722 | AsGsAsmCuTumCuTumCsGsGsTsTsmCsmCsGsA-GN3 | 7184.3 | 7184.0 | 95.9 |
| 1506052 | mCsTsTsmCsCsAsmCsAsGsTsAsTsAsTsmCsT-GN3 | 6890.1 | 6889.6 | 89.5 |
| 1505720 | mCsTsTsmCuCuAumCuAsGsTsAsTsAsTsmCsT-GN3 | 7118.3 | 7118.0 | 93.2 |
| 1620648 | AsGsmCsAsCsTsTsTsAsTsTsGsAsGsTsT-GN3 | 6943.1 | 6942.6 | 90.6 |
| 1525924 | AsGsmCsAumCuTuTuTsAsTsTsGsAsGsTsT-GN3 | 7171.3 | 7170.8 | 93.2 |
| 1523449 | TumCumCsmCsAsTsTsTsmCsAsGsGsAsGsAsmCsmCsTuGuG | 7171.0 | 7170.0 | 90.6 |
| 1523450 | TsmCsmCsmCuAuTuTsTsmCsAsGsGsAsGsAsmCsmCsTsGsG | 7110.0 | 7108.8 | 91.6 |
| 1523451 | TsmCsmCsmCsAsTsTsTsmCsAsGsGsAsGsAumCumCuTsGsG | 7110.0 | 7108.8 | 90.7 |
| 1523452 | mCuAumCsTsTsTsTsAsmCsAsTsTsAsGuGuA | 5634.7 | 5633.7 | 85.9 |
| 1523453 | mCsAsmCsTuTuTuTsAsmCsAsTsTsAsGsGsA | 5573.6 | 5572.5 | 90.3 |
| 1523454 | mCsAsmCsTsTsTsTsAsmCsAsTuTuAuGsGsA | 5573.6 | 5572.8 | 90.4 |
| 1523455 | TumCuTsmCsAsTsTsTsAsTsTsTsmCsGuGumC | 5606.6 | 5605.5 | 86.1 |
| 1523456 | TsmCsTsmCuAuTuTsTsAsTsTsTsmCsGsGsmC | 5545.7 | 5544.6 | 86.1 |
| 1523457 | TsmCsTsmCsAsTsTsTsAsTsTuTumCuGsGsmC | 5545.7 | 5544.6 | 92.4 |
Supplementary Figure 2. Analytical data for synthesized ASOs

## Slide 4
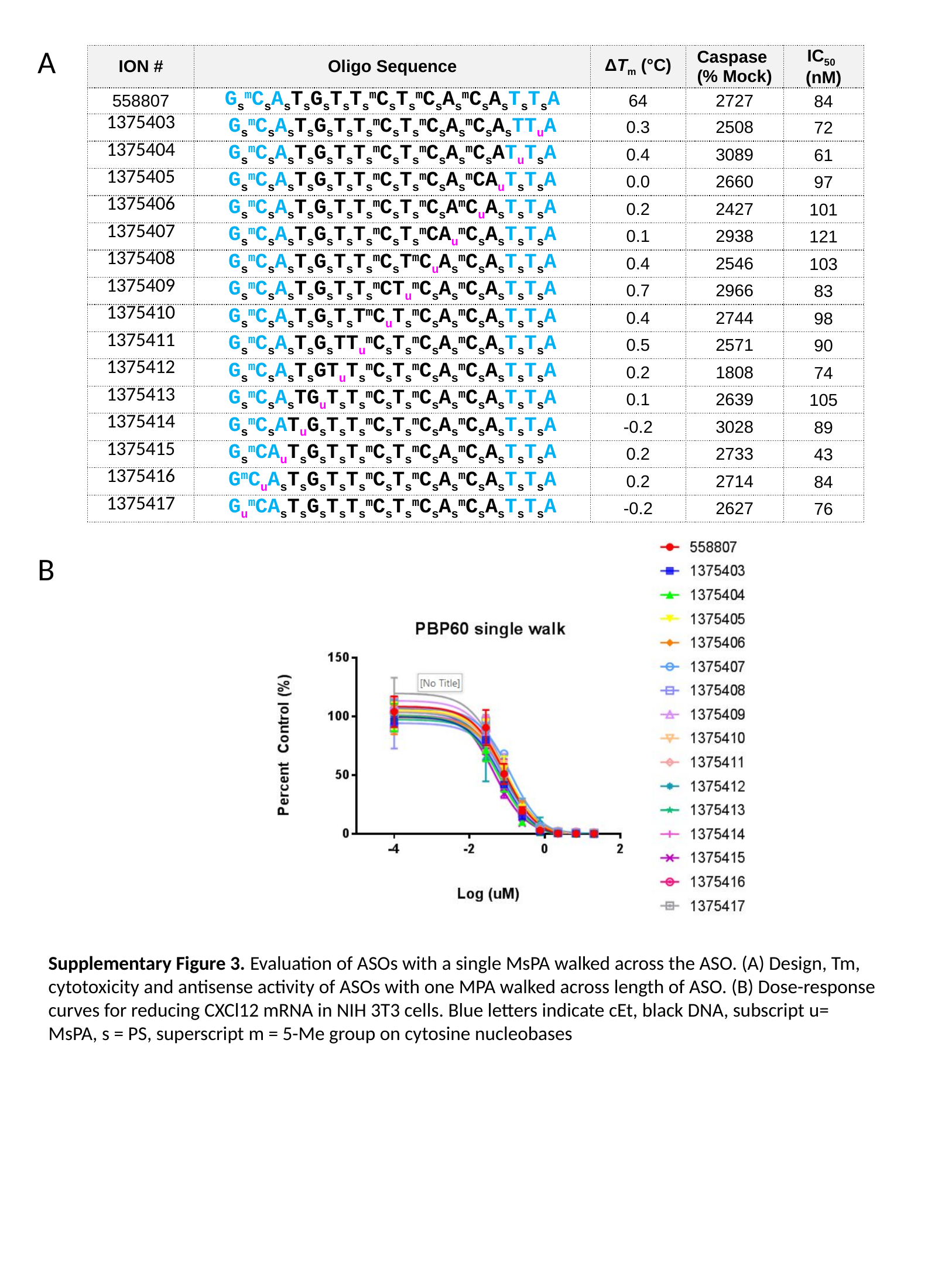

A
| ION # | Oligo Sequence | ΔTm (°C) | Caspase  (% Mock) | IC50  (nM) |
| --- | --- | --- | --- | --- |
| 558807 | GsmCsAsTsGsTsTsmCsTsmCsAsmCsAsTsTsA | 64 | 2727 | 84 |
| 1375403 | GsmCsAsTsGsTsTsmCsTsmCsAsmCsAsTTuA | 0.3 | 2508 | 72 |
| 1375404 | GsmCsAsTsGsTsTsmCsTsmCsAsmCsATuTsA | 0.4 | 3089 | 61 |
| 1375405 | GsmCsAsTsGsTsTsmCsTsmCsAsmCAuTsTsA | 0.0 | 2660 | 97 |
| 1375406 | GsmCsAsTsGsTsTsmCsTsmCsAmCuAsTsTsA | 0.2 | 2427 | 101 |
| 1375407 | GsmCsAsTsGsTsTsmCsTsmCAumCsAsTsTsA | 0.1 | 2938 | 121 |
| 1375408 | GsmCsAsTsGsTsTsmCsTmCuAsmCsAsTsTsA | 0.4 | 2546 | 103 |
| 1375409 | GsmCsAsTsGsTsTsmCTumCsAsmCsAsTsTsA | 0.7 | 2966 | 83 |
| 1375410 | GsmCsAsTsGsTsTmCuTsmCsAsmCsAsTsTsA | 0.4 | 2744 | 98 |
| 1375411 | GsmCsAsTsGsTTumCsTsmCsAsmCsAsTsTsA | 0.5 | 2571 | 90 |
| 1375412 | GsmCsAsTsGTuTsmCsTsmCsAsmCsAsTsTsA | 0.2 | 1808 | 74 |
| 1375413 | GsmCsAsTGuTsTsmCsTsmCsAsmCsAsTsTsA | 0.1 | 2639 | 105 |
| 1375414 | GsmCsATuGsTsTsmCsTsmCsAsmCsAsTsTsA | -0.2 | 3028 | 89 |
| 1375415 | GsmCAuTsGsTsTsmCsTsmCsAsmCsAsTsTsA | 0.2 | 2733 | 43 |
| 1375416 | GmCuAsTsGsTsTsmCsTsmCsAsmCsAsTsTsA | 0.2 | 2714 | 84 |
| 1375417 | GumCAsTsGsTsTsmCsTsmCsAsmCsAsTsTsA | -0.2 | 2627 | 76 |
B
Supplementary Figure 3. Evaluation of ASOs with a single MsPA walked across the ASO. (A) Design, Tm, cytotoxicity and antisense activity of ASOs with one MPA walked across length of ASO. (B) Dose-response curves for reducing CXCl12 mRNA in NIH 3T3 cells. Blue letters indicate cEt, black DNA, subscript u= MsPA, s = PS, superscript m = 5-Me group on cytosine nucleobases

## Slide 5
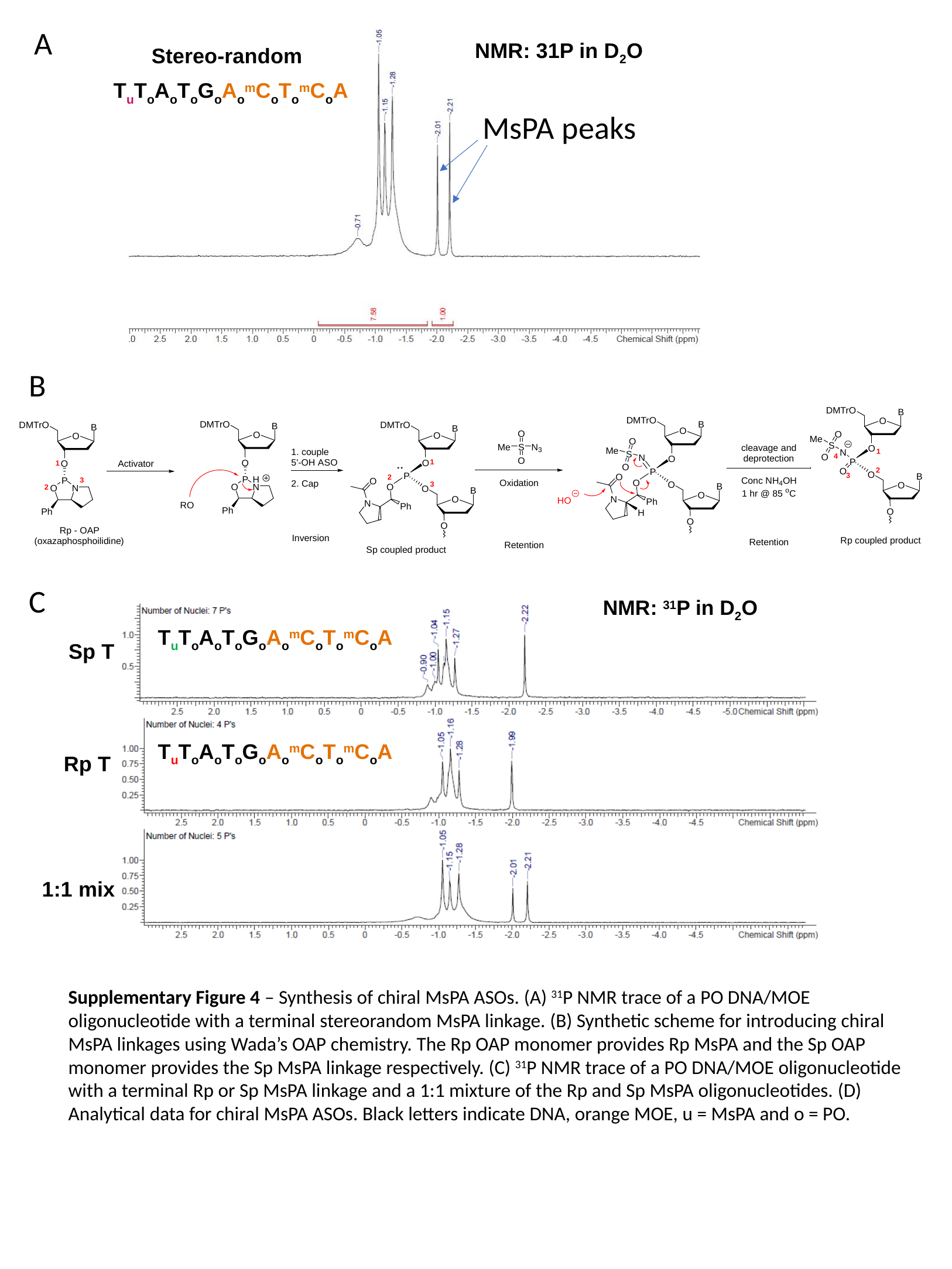

A
NMR: 31P in D2O
Stereo-random
TuToAoToGoAomCoTomCoA
MsPA peaks
B
C
NMR: 31P in D2O
TuToAoToGoAomCoTomCoA
Sp T
TuToAoToGoAomCoTomCoA
Rp T
1:1 mix
Supplementary Figure 4 – Synthesis of chiral MsPA ASOs. (A) 31P NMR trace of a PO DNA/MOE oligonucleotide with a terminal stereorandom MsPA linkage. (B) Synthetic scheme for introducing chiral MsPA linkages using Wada’s OAP chemistry. The Rp OAP monomer provides Rp MsPA and the Sp OAP monomer provides the Sp MsPA linkage respectively. (C) 31P NMR trace of a PO DNA/MOE oligonucleotide with a terminal Rp or Sp MsPA linkage and a 1:1 mixture of the Rp and Sp MsPA oligonucleotides. (D) Analytical data for chiral MsPA ASOs. Black letters indicate DNA, orange MOE, u = MsPA and o = PO.

## Slide 6
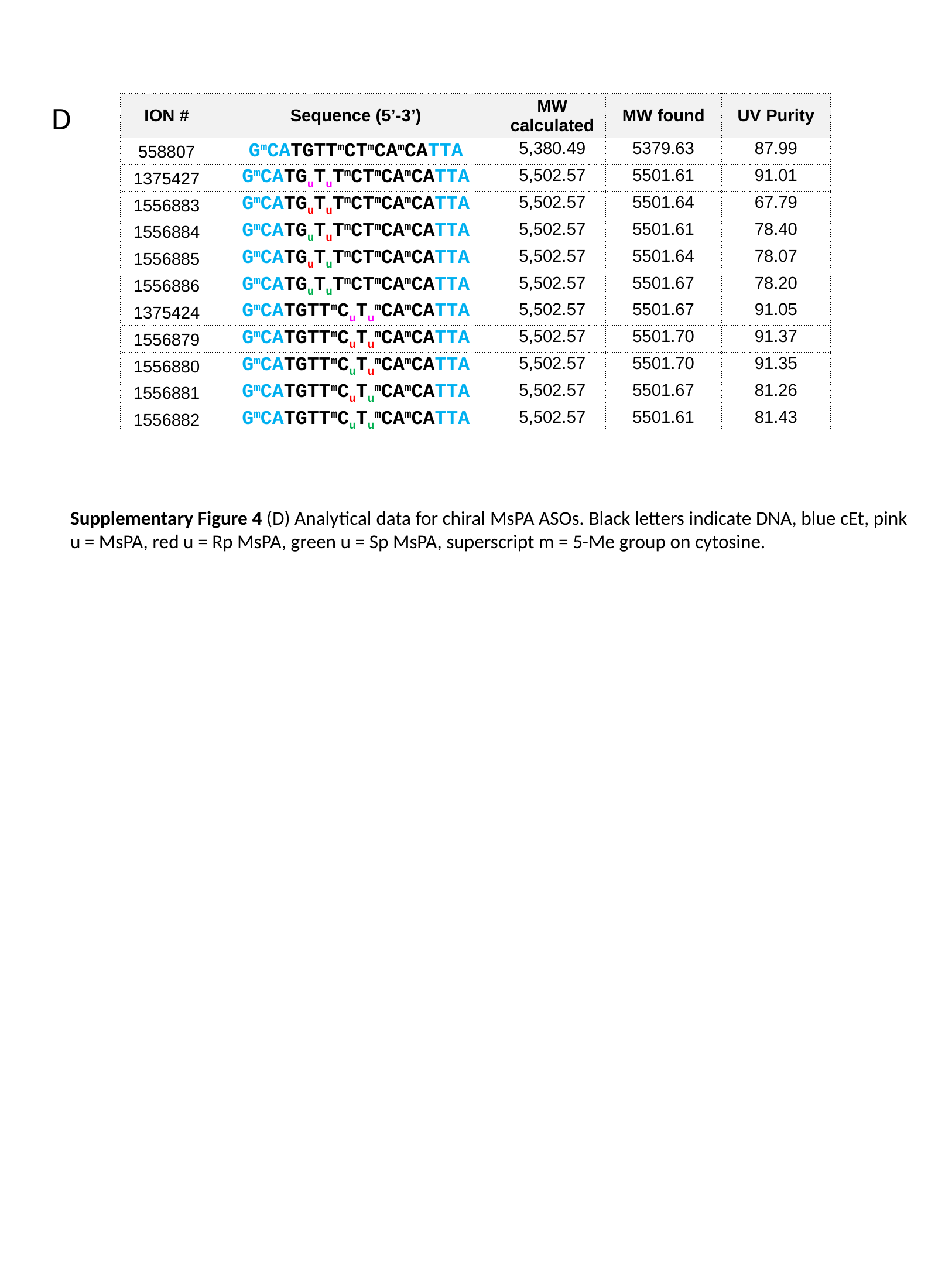

D
| ION # | Sequence (5’-3’) | MW calculated | MW found | UV Purity |
| --- | --- | --- | --- | --- |
| 558807 | GmCATGTTmCTmCAmCATTA | 5,380.49 | 5379.63 | 87.99 |
| 1375427 | GmCATGuTuTmCTmCAmCATTA | 5,502.57 | 5501.61 | 91.01 |
| 1556883 | GmCATGuTuTmCTmCAmCATTA | 5,502.57 | 5501.64 | 67.79 |
| 1556884 | GmCATGuTuTmCTmCAmCATTA | 5,502.57 | 5501.61 | 78.40 |
| 1556885 | GmCATGuTuTmCTmCAmCATTA | 5,502.57 | 5501.64 | 78.07 |
| 1556886 | GmCATGuTuTmCTmCAmCATTA | 5,502.57 | 5501.67 | 78.20 |
| 1375424 | GmCATGTTmCuTumCAmCATTA | 5,502.57 | 5501.67 | 91.05 |
| 1556879 | GmCATGTTmCuTumCAmCATTA | 5,502.57 | 5501.70 | 91.37 |
| 1556880 | GmCATGTTmCuTumCAmCATTA | 5,502.57 | 5501.70 | 91.35 |
| 1556881 | GmCATGTTmCuTumCAmCATTA | 5,502.57 | 5501.67 | 81.26 |
| 1556882 | GmCATGTTmCuTumCAmCATTA | 5,502.57 | 5501.61 | 81.43 |
Supplementary Figure 4 (D) Analytical data for chiral MsPA ASOs. Black letters indicate DNA, blue cEt, pink u = MsPA, red u = Rp MsPA, green u = Sp MsPA, superscript m = 5-Me group on cytosine.

## Slide 7
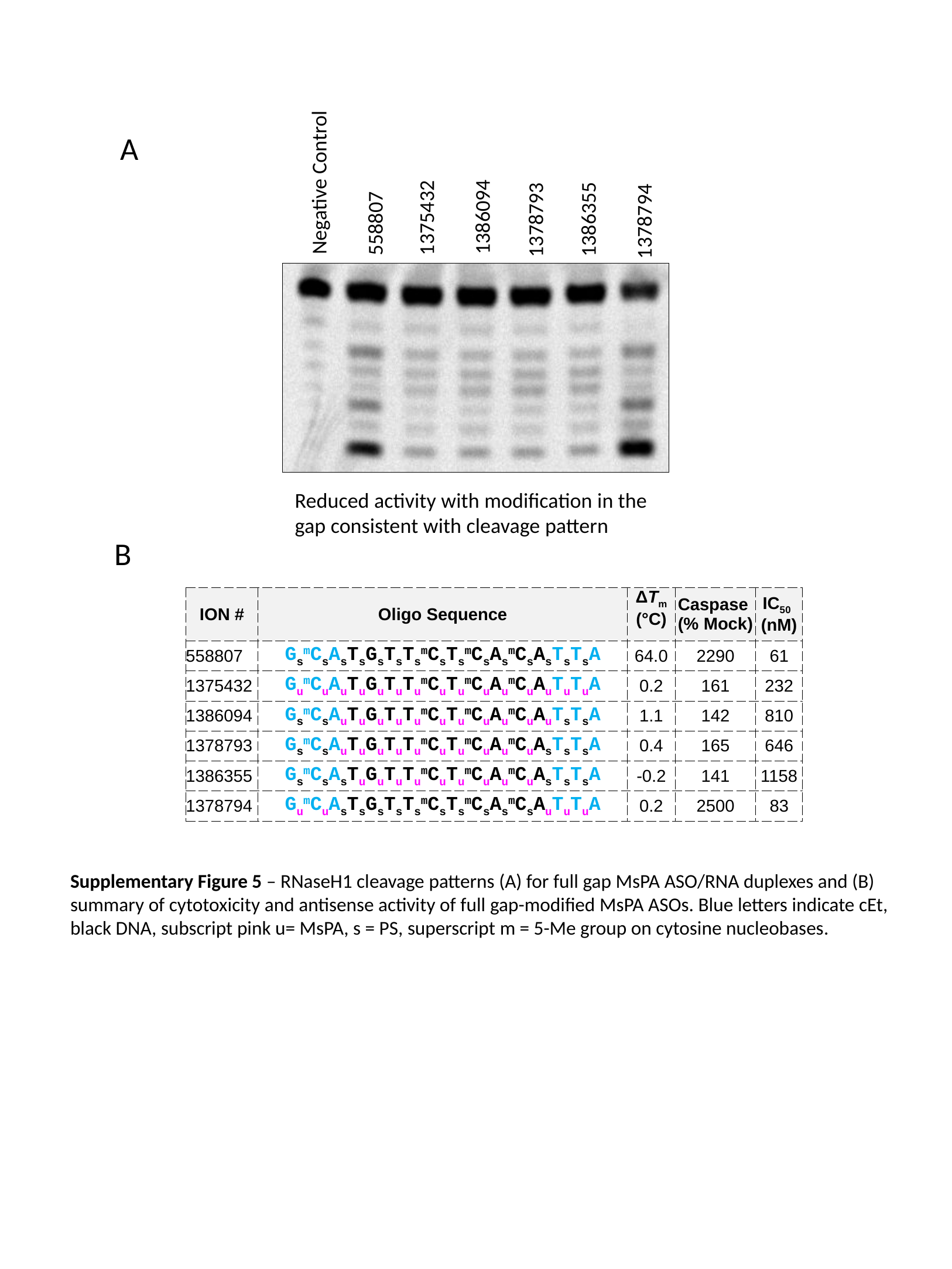

A
Negative Control
1386094
1375432
1386355
1378793
1378794
558807
Reduced activity with modification in the gap consistent with cleavage pattern
B
| ION # | Oligo Sequence | ΔTm (°C) | Caspase (% Mock) | IC50 (nM) |
| --- | --- | --- | --- | --- |
| 558807 | GsmCsAsTsGsTsTsmCsTsmCsAsmCsAsTsTsA | 64.0 | 2290 | 61 |
| 1375432 | GumCuAuTuGuTuTumCuTumCuAumCuAuTuTuA | 0.2 | 161 | 232 |
| 1386094 | GsmCsAuTuGuTuTumCuTumCuAumCuAuTsTsA | 1.1 | 142 | 810 |
| 1378793 | GsmCsAuTuGuTuTumCuTumCuAumCuAsTsTsA | 0.4 | 165 | 646 |
| 1386355 | GsmCsAsTuGuTuTumCuTumCuAumCuAsTsTsA | -0.2 | 141 | 1158 |
| 1378794 | GumCuAsTsGsTsTsmCsTsmCsAsmCsAuTuTuA | 0.2 | 2500 | 83 |
Supplementary Figure 5 – RNaseH1 cleavage patterns (A) for full gap MsPA ASO/RNA duplexes and (B) summary of cytotoxicity and antisense activity of full gap-modified MsPA ASOs. Blue letters indicate cEt, black DNA, subscript pink u= MsPA, s = PS, superscript m = 5-Me group on cytosine nucleobases.

## Slide 8
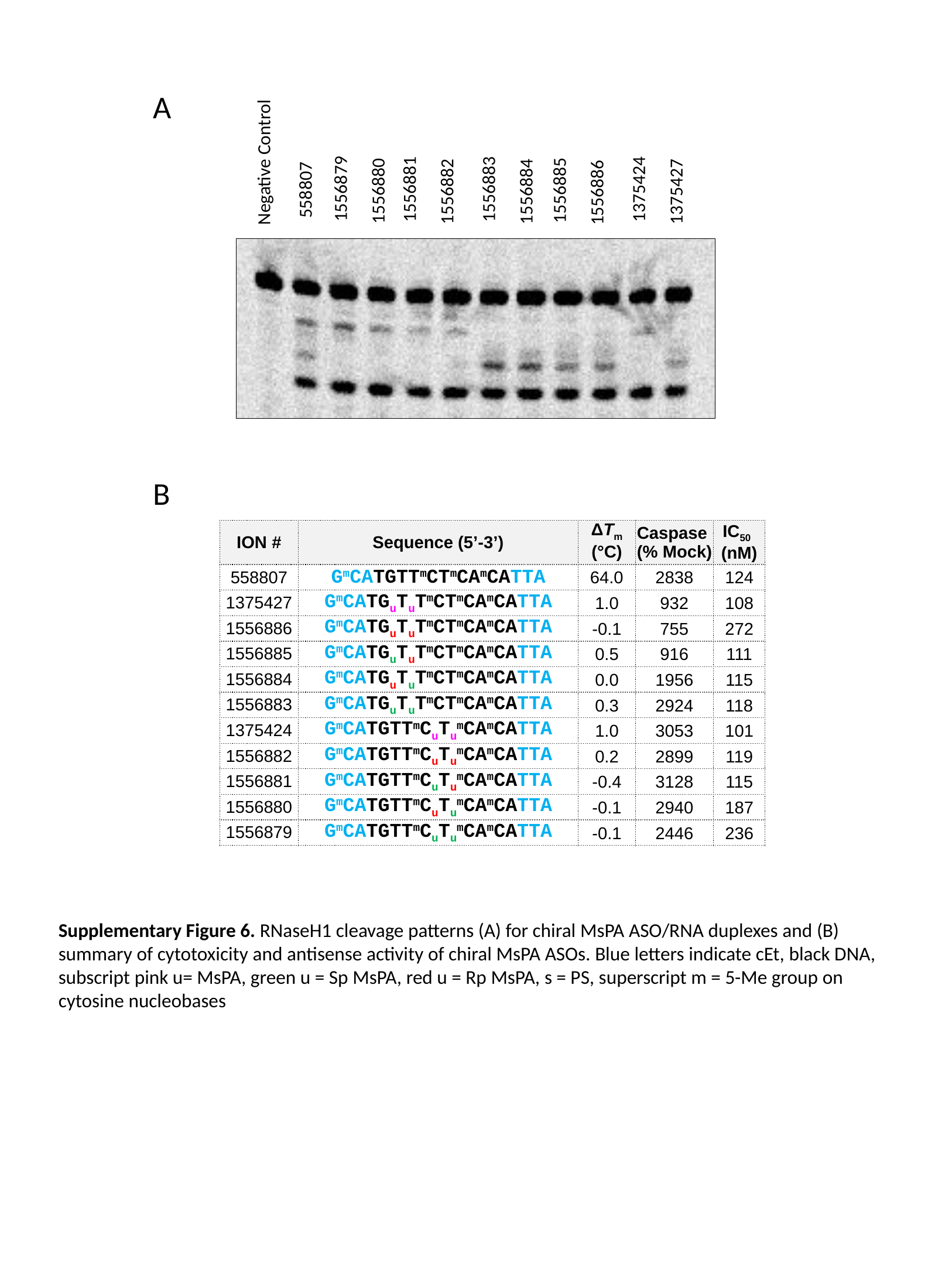

A
Negative Control
1556879
1556881
1556883
1375424
558807
1556885
1556880
1556882
1556884
1375427
1556886
B
| ION # | Sequence (5’-3’) | ΔTm (°C) | Caspase  (% Mock) | IC50  (nM) |
| --- | --- | --- | --- | --- |
| 558807 | GmCATGTTmCTmCAmCATTA | 64.0 | 2838 | 124 |
| 1375427 | GmCATGuTuTmCTmCAmCATTA | 1.0 | 932 | 108 |
| 1556886 | GmCATGuTuTmCTmCAmCATTA | -0.1 | 755 | 272 |
| 1556885 | GmCATGuTuTmCTmCAmCATTA | 0.5 | 916 | 111 |
| 1556884 | GmCATGuTuTmCTmCAmCATTA | 0.0 | 1956 | 115 |
| 1556883 | GmCATGuTuTmCTmCAmCATTA | 0.3 | 2924 | 118 |
| 1375424 | GmCATGTTmCuTumCAmCATTA | 1.0 | 3053 | 101 |
| 1556882 | GmCATGTTmCuTumCAmCATTA | 0.2 | 2899 | 119 |
| 1556881 | GmCATGTTmCuTumCAmCATTA | -0.4 | 3128 | 115 |
| 1556880 | GmCATGTTmCuTumCAmCATTA | -0.1 | 2940 | 187 |
| 1556879 | GmCATGTTmCuTumCAmCATTA | -0.1 | 2446 | 236 |
Supplementary Figure 6. RNaseH1 cleavage patterns (A) for chiral MsPA ASO/RNA duplexes and (B) summary of cytotoxicity and antisense activity of chiral MsPA ASOs. Blue letters indicate cEt, black DNA, subscript pink u= MsPA, green u = Sp MsPA, red u = Rp MsPA, s = PS, superscript m = 5-Me group on cytosine nucleobases

## Slide 9
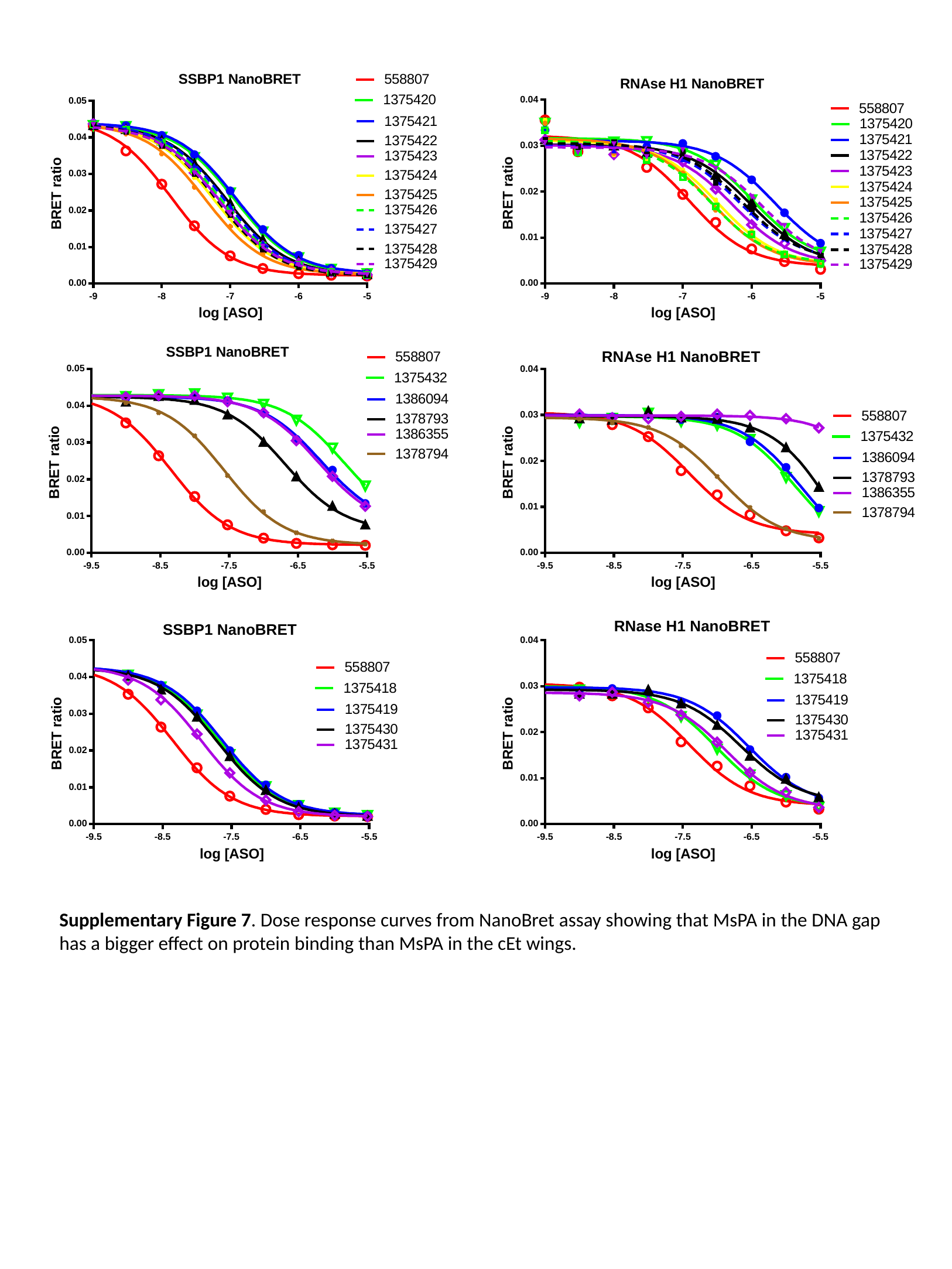

Supplementary Figure 7. Dose response curves from NanoBret assay showing that MsPA in the DNA gap has a bigger effect on protein binding than MsPA in the cEt wings.

## Slide 10
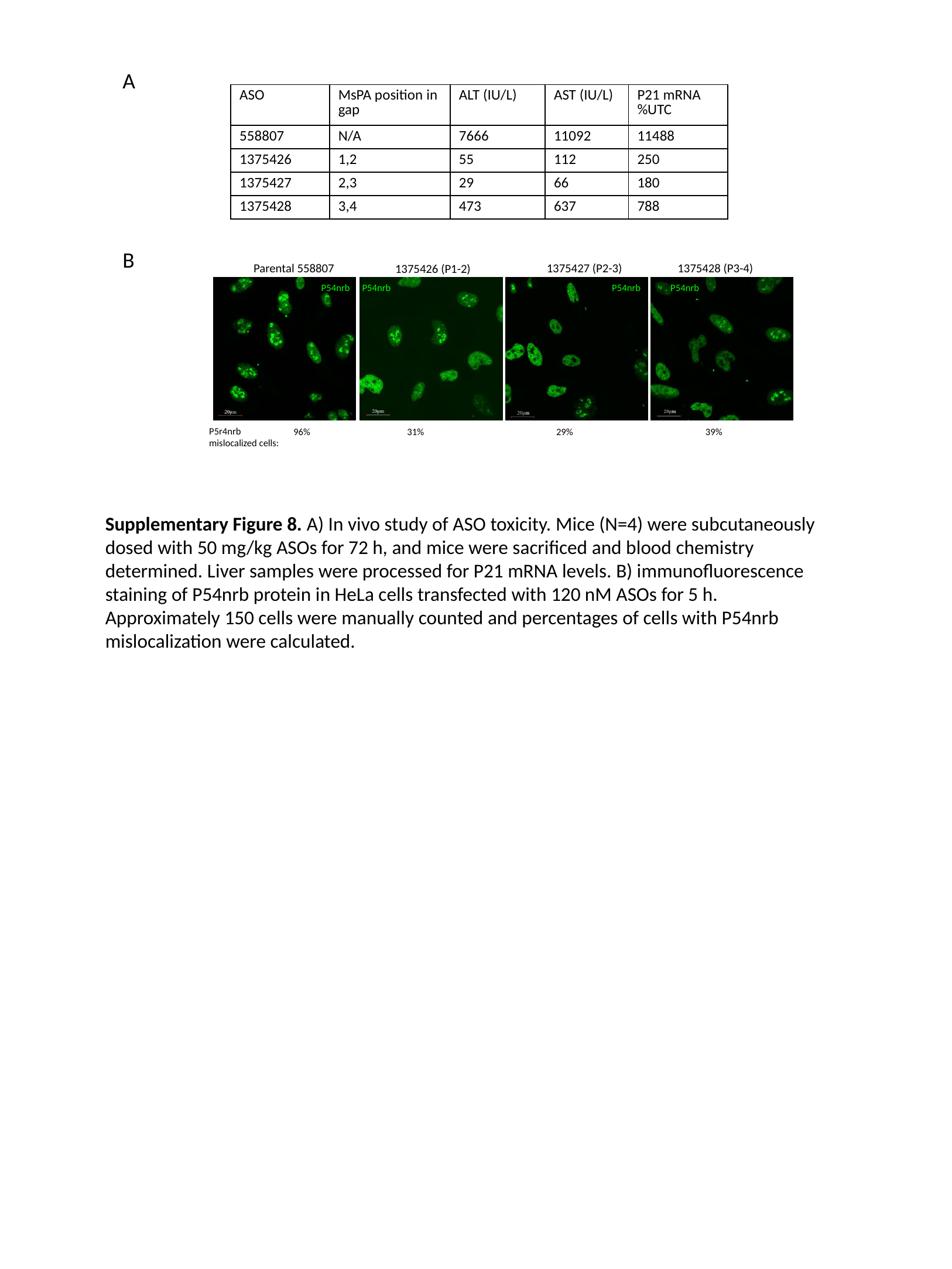

A
| ASO | MsPA position in gap | ALT (IU/L) | AST (IU/L) | P21 mRNA %UTC |
| --- | --- | --- | --- | --- |
| 558807 | N/A | 7666 | 11092 | 11488 |
| 1375426 | 1,2 | 55 | 112 | 250 |
| 1375427 | 2,3 | 29 | 66 | 180 |
| 1375428 | 3,4 | 473 | 637 | 788 |
B
1375427 (P2-3)
1375428 (P3-4)
Parental 558807
1375426 (P1-2)
P54nrb
P54nrb
P54nrb
P54nrb
P5r4nrb
mislocalized cells:
96%
31%
29%
39%
Supplementary Figure 8. A) In vivo study of ASO toxicity. Mice (N=4) were subcutaneously dosed with 50 mg/kg ASOs for 72 h, and mice were sacrificed and blood chemistry determined. Liver samples were processed for P21 mRNA levels. B) immunofluorescence staining of P54nrb protein in HeLa cells transfected with 120 nM ASOs for 5 h. Approximately 150 cells were manually counted and percentages of cells with P54nrb mislocalization were calculated.

## Slide 11
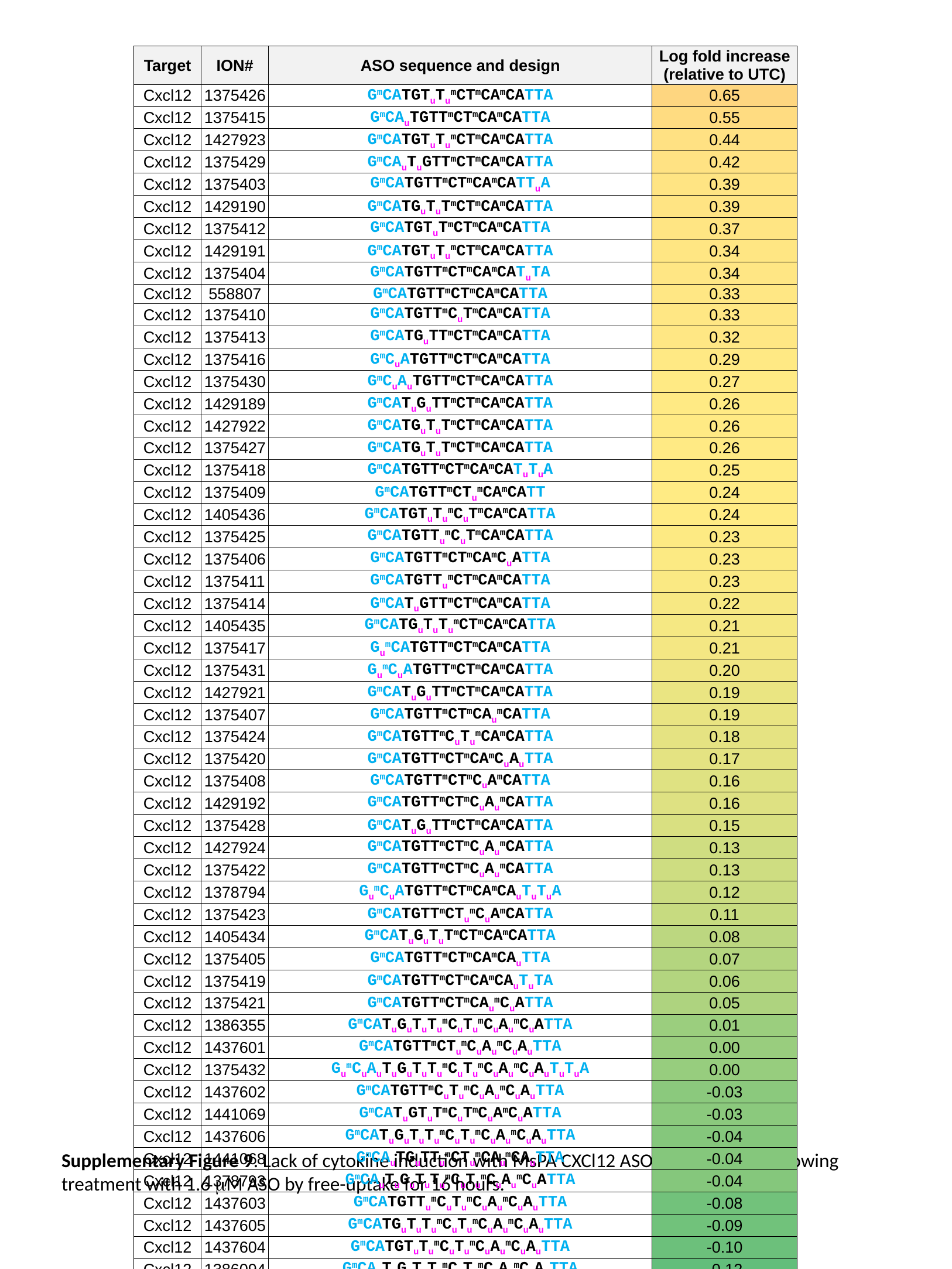

| Target | ION# | ASO sequence and design | Log fold increase (relative to UTC) |
| --- | --- | --- | --- |
| Cxcl12 | 1375426 | GmCATGTuTumCTmCAmCATTA | 0.65 |
| Cxcl12 | 1375415 | GmCAuTGTTmCTmCAmCATTA | 0.55 |
| Cxcl12 | 1427923 | GmCATGTuTumCTmCAmCATTA | 0.44 |
| Cxcl12 | 1375429 | GmCAuTuGTTmCTmCAmCATTA | 0.42 |
| Cxcl12 | 1375403 | GmCATGTTmCTmCAmCATTuA | 0.39 |
| Cxcl12 | 1429190 | GmCATGuTuTmCTmCAmCATTA | 0.39 |
| Cxcl12 | 1375412 | GmCATGTuTmCTmCAmCATTA | 0.37 |
| Cxcl12 | 1429191 | GmCATGTuTumCTmCAmCATTA | 0.34 |
| Cxcl12 | 1375404 | GmCATGTTmCTmCAmCATuTA | 0.34 |
| Cxcl12 | 558807 | GmCATGTTmCTmCAmCATTA | 0.33 |
| Cxcl12 | 1375410 | GmCATGTTmCuTmCAmCATTA | 0.33 |
| Cxcl12 | 1375413 | GmCATGuTTmCTmCAmCATTA | 0.32 |
| Cxcl12 | 1375416 | GmCuATGTTmCTmCAmCATTA | 0.29 |
| Cxcl12 | 1375430 | GmCuAuTGTTmCTmCAmCATTA | 0.27 |
| Cxcl12 | 1429189 | GmCATuGuTTmCTmCAmCATTA | 0.26 |
| Cxcl12 | 1427922 | GmCATGuTuTmCTmCAmCATTA | 0.26 |
| Cxcl12 | 1375427 | GmCATGuTuTmCTmCAmCATTA | 0.26 |
| Cxcl12 | 1375418 | GmCATGTTmCTmCAmCATuTuA | 0.25 |
| Cxcl12 | 1375409 | GmCATGTTmCTumCAmCATT | 0.24 |
| Cxcl12 | 1405436 | GmCATGTuTumCuTmCAmCATTA | 0.24 |
| Cxcl12 | 1375425 | GmCATGTTumCuTmCAmCATTA | 0.23 |
| Cxcl12 | 1375406 | GmCATGTTmCTmCAmCuATTA | 0.23 |
| Cxcl12 | 1375411 | GmCATGTTumCTmCAmCATTA | 0.23 |
| Cxcl12 | 1375414 | GmCATuGTTmCTmCAmCATTA | 0.22 |
| Cxcl12 | 1405435 | GmCATGuTuTumCTmCAmCATTA | 0.21 |
| Cxcl12 | 1375417 | GumCATGTTmCTmCAmCATTA | 0.21 |
| Cxcl12 | 1375431 | GumCuATGTTmCTmCAmCATTA | 0.20 |
| Cxcl12 | 1427921 | GmCATuGuTTmCTmCAmCATTA | 0.19 |
| Cxcl12 | 1375407 | GmCATGTTmCTmCAumCATTA | 0.19 |
| Cxcl12 | 1375424 | GmCATGTTmCuTumCAmCATTA | 0.18 |
| Cxcl12 | 1375420 | GmCATGTTmCTmCAmCuAuTTA | 0.17 |
| Cxcl12 | 1375408 | GmCATGTTmCTmCuAmCATTA | 0.16 |
| Cxcl12 | 1429192 | GmCATGTTmCTmCuAumCATTA | 0.16 |
| Cxcl12 | 1375428 | GmCATuGuTTmCTmCAmCATTA | 0.15 |
| Cxcl12 | 1427924 | GmCATGTTmCTmCuAumCATTA | 0.13 |
| Cxcl12 | 1375422 | GmCATGTTmCTmCuAumCATTA | 0.13 |
| Cxcl12 | 1378794 | GumCuATGTTmCTmCAmCAuTuTuA | 0.12 |
| Cxcl12 | 1375423 | GmCATGTTmCTumCuAmCATTA | 0.11 |
| Cxcl12 | 1405434 | GmCATuGuTuTmCTmCAmCATTA | 0.08 |
| Cxcl12 | 1375405 | GmCATGTTmCTmCAmCAuTTA | 0.07 |
| Cxcl12 | 1375419 | GmCATGTTmCTmCAmCAuTuTA | 0.06 |
| Cxcl12 | 1375421 | GmCATGTTmCTmCAumCuATTA | 0.05 |
| Cxcl12 | 1386355 | GmCATuGuTuTumCuTumCuAumCuATTA | 0.01 |
| Cxcl12 | 1437601 | GmCATGTTmCTumCuAumCuAuTTA | 0.00 |
| Cxcl12 | 1375432 | GumCuAuTuGuTuTumCuTumCuAumCuAuTuTuA | 0.00 |
| Cxcl12 | 1437602 | GmCATGTTmCuTumCuAumCuAuTTA | -0.03 |
| Cxcl12 | 1441069 | GmCATuGTuTmCuTmCuAmCuATTA | -0.03 |
| Cxcl12 | 1437606 | GmCATuGuTuTumCuTumCuAumCuAuTTA | -0.04 |
| Cxcl12 | 1441068 | GmCAuTGuTTumCTumCAumCAuTTA | -0.04 |
| Cxcl12 | 1378793 | GmCAuTuGuTuTumCuTumCuAumCuATTA | -0.04 |
| Cxcl12 | 1437603 | GmCATGTTumCuTumCuAumCuAuTTA | -0.08 |
| Cxcl12 | 1437605 | GmCATGuTuTumCuTumCuAumCuAuTTA | -0.09 |
| Cxcl12 | 1437604 | GmCATGTuTumCuTumCuAumCuAuTTA | -0.10 |
| Cxcl12 | 1386094 | GmCAuTuGuTuTumCuTumCuAumCuAuTTA | -0.12 |
Supplementary Figure 9. Lack of cytokine induction with MsPA CXCl12 ASOs in BJAB cells following treatment with 1.6 mM ASO by free-uptake for 16 hours.

## Slide 12
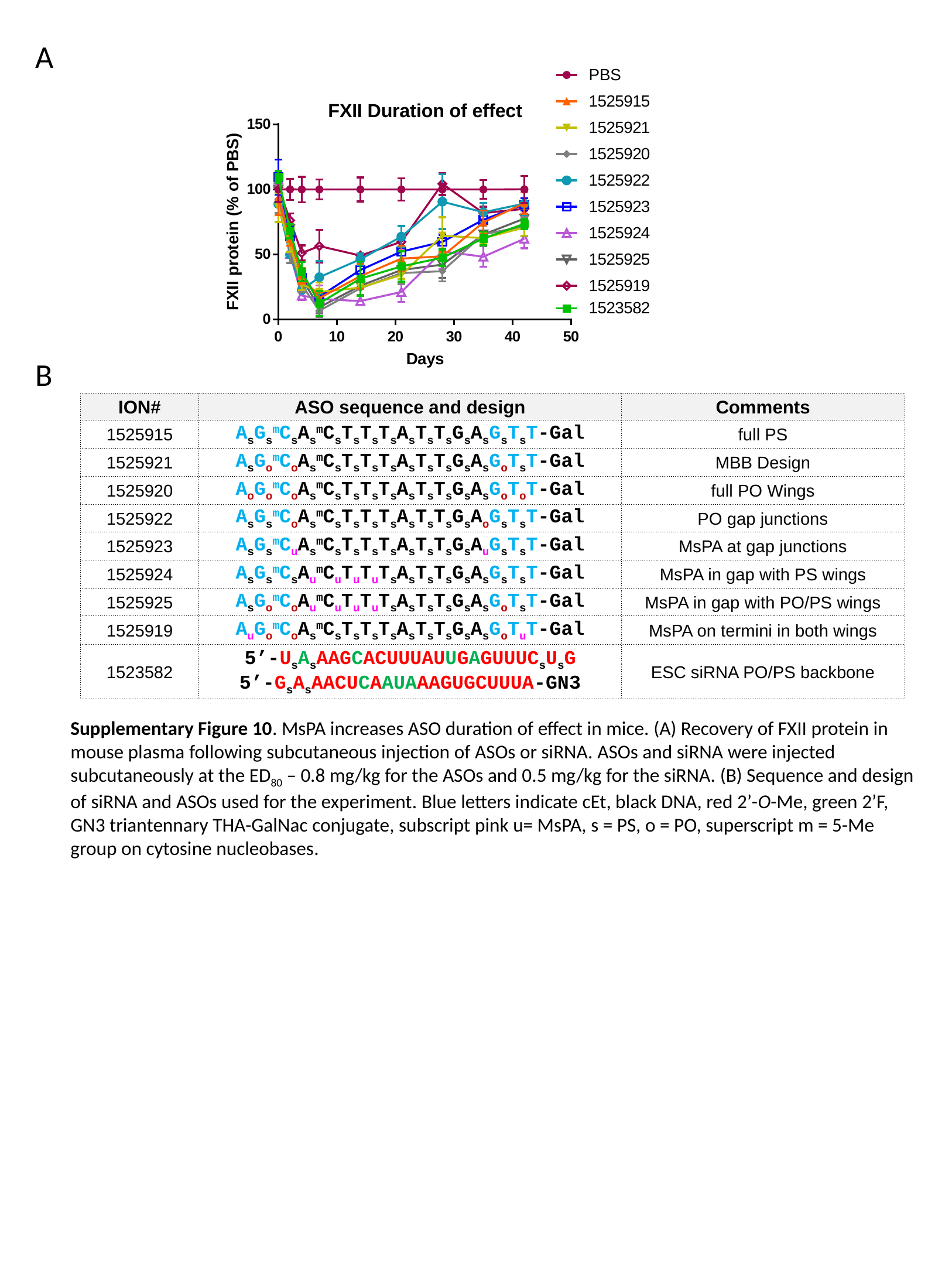

A
B
| ION# | ASO sequence and design | Comments |
| --- | --- | --- |
| 1525915 | AsGsmCsAsmCsTsTsTsAsTsTsGsAsGsTsT-Gal | full PS |
| 1525921 | AsGomCoAsmCsTsTsTsAsTsTsGsAsGoTsT-Gal | MBB Design |
| 1525920 | AoGomCoAsmCsTsTsTsAsTsTsGsAsGoToT-Gal | full PO Wings |
| 1525922 | AsGsmCoAsmCsTsTsTsAsTsTsGsAoGsTsT-Gal | PO gap junctions |
| 1525923 | AsGsmCuAsmCsTsTsTsAsTsTsGsAuGsTsT-Gal | MsPA at gap junctions |
| 1525924 | AsGsmCsAumCuTuTuTsAsTsTsGsAsGsTsT-Gal | MsPA in gap with PS wings |
| 1525925 | AsGomCoAumCuTuTuTsAsTsTsGsAsGoTsT-Gal | MsPA in gap with PO/PS wings |
| 1525919 | AuGomCoAsmCsTsTsTsAsTsTsGsAsGoTuT-Gal | MsPA on termini in both wings |
| 1523582 | 5’-UsAsAAGCACUUUAUUGAGUUUCsUsG 5’-GsAsAACUCAAUAAAGUGCUUUA-GN3 | ESC siRNA PO/PS backbone |
Supplementary Figure 10. MsPA increases ASO duration of effect in mice. (A) Recovery of FXII protein in mouse plasma following subcutaneous injection of ASOs or siRNA. ASOs and siRNA were injected subcutaneously at the ED80 – 0.8 mg/kg for the ASOs and 0.5 mg/kg for the siRNA. (B) Sequence and design of siRNA and ASOs used for the experiment. Blue letters indicate cEt, black DNA, red 2’-O-Me, green 2’F, GN3 triantennary THA-GalNac conjugate, subscript pink u= MsPA, s = PS, o = PO, superscript m = 5-Me group on cytosine nucleobases.
